# NLRC4 Acts as a Non‐Canonical Tumour Suppressor in Lung Adenocarcinoma by Inhibiting PI3K/AKT/mTOR Signalling to Induce Protective Autophagy

**DOI:** 10.1111/jcmm.71420

**Published:** 2026-10-26

**Authors:** Yuxin Zhang, Shuangteng Liu, Rui Li, Mengyu Zhang, Guang Yang, Xinmiao Zhu, Yiqing Qu

**Affiliations:** ^1^ Laboratory of Basic Medical Sciences, Department of Pulmonary and Critical Care Medicine Qilu Hospital of Shandong University Jinan China

**Keywords:** autophagy, lung adenocarcinoma, NLRC4, PI3K/AKT signalling, prognosis

## Abstract

Autophagy plays complex, context‐dependent roles in lung adenocarcinoma (LUAD), but the upstream molecular drivers that govern this process remain elusive. NLRC4 is classically defined as a pyroptosis‐inducing inflammasome sensor, yet its non‐canonical, tumour‐intrinsic function in LUAD progression remains largely unexplored. Through multi‐omics screening and prognostic modelling across TCGA and GEO datasets, we identified NLRC4 as a critical autophagy‐associated protective factor, which was validated in an institutional cohort of paired LUAD tissues. In vitro and in vivo assays, autophagic flux monitoring, reciprocal co‐immunoprecipitation and genetic rescue experiments were employed to elucidate the underlying mechanism. NLRC4 protein was markedly downregulated in LUAD tissues alongside the tumour suppressor PTEN, correlating with advanced TNM stage and poor overall survival. Functionally, restoring NLRC4 expression inhibited LUAD cell proliferation, motility, invasion and epithelial–mesenchymal transition in vitro, whereas NLRC4 depletion accelerated xenograft tumour growth in vivo. Crucially, NLRC4 selectively stimulated complete autophagic flux without triggering Caspase‐1/Gasdermin cleavage or lytic pyroptotic death. Mechanistically, NLRC4 physically interacted with NFKB1 (p105/p50) in a tumour‐selective manner, relieving NF‐κB‐mediated transcriptional repression of PTEN. This restored PTEN expression, thereby tonically dampening the PI3K/AKT/mTOR signalling cascade. Genetic blockade of autophagy (siATG5) or PTEN ablation largely reversed the tumour‐suppressive effects of NLRC4, confirming the functional dependency on the PTEN–autophagy axis. Our study establishes a non‐canonical, tumour‐intrinsic role for NLRC4 in restraining LUAD progression via the PI3K/AKT/mTOR axis to induce protective autophagy, positioning it as a promising prognostic biomarker and potential therapeutic target.

## 1 Introduction

Lung cancer is the most common cancer worldwide and the leading cause of cancer‐related deaths [1]. Non‐small cell lung cancer (NSCLC) constitutes approximately 85% of all lung cancer cases, with lung adenocarcinoma (LUAD) being the most prevalent subtype [2]. Despite advances in treatment, the overall survival rate for LUAD patients remains unsatisfactory, underscoring the need to elucidate the molecular mechanisms driving its progression.

Autophagy is a critical intracellular degradation system that transports and degrades cytoplasmic components via lysosomal pathways [3]. It plays dual roles in cancer: acting as a tumour‐suppressive mechanism in early carcinogenesis but often being co‐opted for tumour cell survival in established malignancies [4, 5]. Multiple signalling pathways regulate autophagy, among which the PI3K/AKT/mTOR axis is one of the most well‐established and frequently dysregulated in cancer [6, 7]. As the primary lipid phosphatase antagonizing PI3K activation, the tumour suppressor PTEN acts as an indispensable upstream gatekeeper that facilitates autophagic induction by dampening PI3K/AKT/mTOR signalling [8, 9].

Several autophagy modulators have been identified in cancer, but the role of NLRC4, a cytoplasmic pattern‐recognition sensor, remains largely unexplored in LUAD. Classically, NLRC4 operates in myeloid immune cells to assemble canonical inflammasomes upon recognizing bacterial ligands, triggering Caspase‐1 activation and lytic pyroptosis [10, 11, 12]. However, emerging evidence indicates that NLR family members often exhibit ‘moonlighting’ functions in non‐myeloid epithelial tissues, acting as transcriptional regulators or signalling scaffolds independent of inflammasome assembly [13, 14, 15, 16]. Given the frequent hyperactivation of PI3K/AKT signalling in LUAD and the non‐canonical versatility of NLR proteins, we hypothesized that NLRC4 may coordinate a tumour‐suppressive signalling network in LUAD by modulating the PTEN/PI3K/AKT‐autophagy axis.

In this study, through multi‐omics screening and prognostic modelling, we identified NLRC4 as a critical autophagy‐associated protective biomarker that is significantly downregulated in LUAD and correlates with favourable clinical outcomes. Functionally, NLRC4 inhibits LUAD proliferation, invasion and epithelial–mesenchymal transition (EMT) in vitro and restrains tumour growth in vivo. Mechanistically, we demonstrate that NLRC4 physically interacts with NFKB1 in a tumour context‐dependent manner, alleviating NF‐κB‐mediated transcriptional repression of PTEN. This de‐repression restores PTEN expression, thereby tonically dampening the PI3K/AKT/mTOR cascade to licence protective autophagic flux independently of canonical pyroptosis. Our findings establish a previously unappreciated non‐canonical role for NLRC4 in LUAD, providing a compelling rationale for leveraging the NLRC4‐PTEN‐autophagy axis in prognostic stratification and therapeutic intervention.

## 2 Methods

### 2.1 Bioinformatic Analysis

RNA sequencing (RNA‐seq) and corresponding clinical data for LUAD were acquired from The Cancer Genome Atlas (TCGA) database (https://portal.gdc.cancer.gov/). Human autophagy‐related genes (ARGs) were retrieved from the Human Autophagy Database (HADb; http://www.autophagy.lu/). Differentially expressed genes (DEGs) were screened using the ‘limma’ package in R with thresholds of |log_2_FC| > 1.0 and false discovery rate (FDR) < 0.01. Prognostic genes were identified via univariate and multivariate Cox proportional hazards regression analyses to establish the risk score model. Multi‐cohort survival validation was performed using independent LUAD datasets from the Gene Expression Omnibus (GEO) database (GSE50081, GSE31210, GSE11969 and GSE68465). Pan‐cancer expression and clinicopathological associations were evaluated via TIMER 2.0 and GEPIA 2.0. Protein–protein interaction (PPI) networks were predicted using the STRING database (v11.5; https://string‐db.org/) with a high‐confidence score cut‐off of 0.70. Correlation between NLRC4 and PTEN expression was cross‐validated using the cBioPortal for Cancer Genomics (https://www.cbioportal.org/). Gene Set Enrichment Analysis (GSEA), Kyoto Encyclopedia of Genes and Genomes (KEGG) and Gene Ontology (GO) pathway analyses were conducted using the ‘clusterProfiler’ R package.

### 2.2 Patient Samples and Cell Lines

Thirty paired fresh‐frozen LUAD tissues and matched adjacent non‐tumour lung tissues were obtained from patients undergoing surgical resection at Qilu Hospital of Shandong University. None of the patients had received preoperative chemotherapy or radiotherapy. Ethical approval was granted by the Medical Ethics Committee of Qilu Hospital, Shandong University (Approval No. KYLL‐2021(KS)‐521), and written informed consent was obtained from all participants.

Human LUAD cell lines (A549, H1975, PC9, H1299), normal human bronchial epithelial cells (BEAS‐2B) and human embryonic kidney cells (HEK‐293T) were purchased from Procell Life Science & Technology Co. Ltd. (Wuhan, China). A549 and PC9 cells were maintained in RPMI‐1640 medium; H1299, H1975 and HEK‐293T cells were cultured in Dulbecco's Modified Eagle Medium (DMEM; Gibco, Thermo Fisher Scientific). BEAS‐2B cells were cultured in Bronchial Epithelial Cell Growth Medium (BEGM; Lonza). All media were supplemented with 10% foetal bovine serum (FBS; Gibco) and 1% penicillin–streptomycin (HyClone). Cells were incubated at 37°C in a humidified atmosphere with 5% CO_2_.

### 2.3 Plasmids, siRNA Transfection and Pharmacological Treatments

For gain‐of‐function studies, full‐length human NLRC4, NFKB1 and PTEN cDNAs were cloned into the pcDNA3.1 vector to generate Flag‐tagged NLRC4 (pcDNA3.1‐3 × Flag‐NLRC4), HA‐tagged NFKB1 (pcDNA3.1‐HA‐NFKB1) and PTEN overexpression (pcDNA3.1‐PTEN) plasmids (GeneChem, Shanghai, China). For loss‐of‐function studies, specific small interfering RNAs (siRNAs) targeting NLRC4 (siNLRC4), ATG5 (siATG5) and PTEN (siPTEN), alongside non‐targeting control siRNAs (siNC), were synthesized by RiboBio (Guangzhou, China). Plasmids and siRNAs were transfected into cells at 60%–80% confluence using Lipofectamine 2000 (Invitrogen, Carlsbad, CA, USA) according to the manufacturer's protocol. Stable knockdown in PC9 cells was established using lentiviral vectors encoding two independent shRNAs targeting NLRC4 (shNLRC4‐1 and shNLRC4‐2) or a non‐targeting scramble control (shNC) (GeneChem), followed by selection with puromycin (2 μg/mL) for 2 weeks. All siRNA sequences are provided in Table S1.

Pharmacological agents were administered as follows: chloroquine (CQ; 20 μM, 24 h; MedChemExpress, HY‐17589A), rapamycin (Rapa; 100 nM, 24 h; MCE, HY‐10219), SC79 (10 μM, 6 h; MCE, HY‐18749), LY294002 (20 μM, 6 h; MCE, HY‐10108) and Bafilomycin A1 (Baf A1; 100 nM, 4 h; MCE, HY‐100558). To establish technical positive controls for canonical pyroptosis, cells were primed with lipopolysaccharide (LPS; 1 μg/mL, 4 h; Sigma‐Aldrich, L2630) followed by Nigericin stimulation (10 μM, 1 h; MCE, HY‐100381).

### 2.4 Western Blot Analysis

Cellular lysis was performed using RIPA buffer (Beyotime), which was supplemented with 1% protease and phosphatase inhibitors to facilitate the extraction of proteins. Proteins were isolated using an SDS‐PAGE gel and then moved to a polyvinylidene difluoride (PVDF) membrane via an electrical transfer method. In order to mitigate nonspecific binding of antibodies, the membrane was subjected to a blocking procedure for 1 h using a 5% nonfat milk solution prior to being incubated with primary antibodies overnight at a temperature of 4°C. Following incubation with the appropriate secondary antibodies, the membranes were subsequently analysed utilizing an enhanced chemiluminescence system. All primary antibodies and dilution ratios are listed in Table S2.

### 2.5 RT‐qPCR

Total RNA was extracted from cells using TRIzol reagent (Invitrogen) to generate cDNA for RT‐qPCR analysis. Then, the cDNA was amplified with QuantiTect PCR Kits (Qiagen, Redwood City, USA) using a 7500 Real‐Time PCR System (Applied Biosystems, Foster City, USA). The relative RNA expression was calculated using the 2^−ΔΔCt^ method. GAPDH was used as the control.

### 2.6 Autophagic Flux Monitoring

Cells were seeded in confocal dishes and stably transduced with mRFP‐GFP‐LC3B tandem fluorescent lentivirus (HanBio Therapeutics, Shanghai, China). Following the indicated genetic or pharmacological treatments, cells were fixed with 4% paraformaldehyde and counterstained with DAPI (1 μg/mL; Solarbio) to visualize nuclei. Images were captured using a confocal laser scanning microscope (Germany). Autophagic flux was assessed by quantifying the ratio of red‐only autolysosome puncta (GFP^−^/RFP^+^) to yellow autophagosome puncta (GFP^+^/RFP^+^) per cell (*n* ≥ 30 cells per condition). Net autophagic flux was biochemically validated by measuring ΔLC3B‐II accumulation in the presence and absence of Bafilomycin A1 (100 nM, 4 h).

### 2.7 Transmission Electron Microscopy (TEM)

Cell lines were cultured and harvested at the logarithmic growth phase. The cells were then fixed with 3% glutaraldehyde and 2% paraformaldehyde in 0.1 M cacodylate buffer (pH 7.3) to preserve their morphological structure. After fixation, the cells were washed in 0.1 M sodium cacodylate buffer and treated with 0.1% Millipore‐filtered cacodylate buffered tannic acid, followed by post‐fixation with 1% osmium tetroxide to ensure preservation of cellular lipids. Next, the cells were dehydrated through a graded ethanol series (50%, 70%, 90% and 100%) and then transitioned to acetone. The dehydrated cells were infiltrated with a liquid epoxy resin (Sigma 45,345), which were then polymerized at 60°C for 48 h to form solid blocks. Once the resin blocks were solidified, ultra‐thin sections (about 70–90 nm) were cut using an ultramicrotome and collected onto copper grids. The sections were stained with uranyl acetate and lead citrate to enhance contrast. Finally, the prepared samples were examined using TEM to observe and capture images of the ultrastructural details.

### 2.8 Cell Counting Kit‐8 (CCK‐8) Assay

Cell proliferation and viability were assessed using a Cell Counting Kit‐8 (CCK‐8; APExBIO, Houston, TX, USA, #K1018) in accordance with the manufacturer's instructions. Following indicated genetic manipulations or pharmacological interventions, LUAD cells were harvested, resuspended and seeded into 96‐well plates. At designated time points, 10 μL of CCK‐8 solution was added to each well. The absorbance was quantified at 450 nm utilizing a microplate reader following a 2 h incubation period in the absence of light. The experiment was carried out independently on three separate occasions.

### 2.9 5‐Ethynyl‐2′‐Deoxyuridine (EdU) Assay

Following designated genetic manipulations or pharmacological treatments, LUAD cells were seeded into 96‐well plates. After an overnight culture, EdU staining was performed utilizing the EdU in vitro kit (Beyotime, #C0075S), adhering to the specified protocols. Subsequently, the labelled cells were imaged using a fluorescence microscope. The experiment was carried out independently on three separate occasions.

### 2.10 Wound‐Healing Assay

Cells that were transfected at an appropriate amount were plated in a six‐well plate until they were fully grown in the wells. Subsequently, a linear incision was made in the cell monolayer utilizing a sterile 200 μL pipette tip. Following three washes with phosphate‐buffered saline, the wells were substituted with serum‐free medium. The width of the wound was assessed and quantified using a microscope at the initial time point (0 h) and again after a duration of 24 h. The experiment was carried out independently on three separate occasions.

### 2.11 Transwell Assay

Cells that had been transfected with the requisite amount were introduced into the upper chamber of the transwell apparatus, which contained a serum‐free medium. Conversely, the lower chamber was supplemented with 800 μL of medium enriched with 20% FBS. The cells located in the lower chamber underwent fixation for a duration of 30 min, followed by a staining process lasting 20 min, after which they were visualized using a microscope after 2 days. The experiment was carried out independently on three separate occasions.

### 2.12 Lactate Dehydrogenase (LDH) Release Cytotoxicity Assay

Extracellular LDH release was determined using the LDH Release Assay Kit with WST‐8 (Beyotime, #C0019) according to the manufacturer's instructions. Cell culture supernatants from the indicated treatment groups were collected, and the cleared supernatant was mixed with freshly prepared LDH assay working solution in a 96‐well plate, then incubated at 37°C in the dark for 20 min. The reaction was terminated by adding Stop Solution to each well. The absorbance was quantified at 450 nm utilizing a microplate reader (reference wavelength: 650 nm). Relative LDH release activity was calculated after subtracting the background absorbance of cell‐free medium controls across three independent biological replicates (*n* = 3).

### 2.13 Co‐Immunoprecipitation (Co‐IP) Assays

Approximately 1 × 10^7^ cells were harvested and lysed using RIPA buffer supplemented with protease inhibitors. Five percentage of the cell lysate was set aside as the input control. The remaining cell lysate was then incubated with primary antibody overnight at 4°C. Then the cell lysate was incubated with protein A/G beads for 6 h at 4°C. After the incubation, the bead‐protein complexes were washed three times with NETN buffer to remove non‐specifically bound proteins, ensuring specificity in the subsequent steps. Then the proteins were removed from the beads with a loading buffer under standard denaturing conditions. Purified proteins were detected by Western blotting.

### 2.14 In Vivo Experiments

The female mice provided by VitoLiva Animal Company were randomly assigned to either test or comparison groups to create a xenograft tumour model. Each experimental group contained five mice (*n* = 5). Then, the transfected PC9 cells with the corresponding lentivirus were subcutaneously implanted in the right armpit area. Tumour sizes were assessed every 7 days, and their volumes were determined using the formula: *V* = (length × width^2^)/2. After 28 days, the volume and weight of each tumour were recorded and measured. Tumour volume and weight were measured by investigators blinded to group allocation to reduce assessment bias. The mice were randomly allocated and maintained under specific pathogen‐free conditions throughout the study. All experiments were performed in accordance with the guidelines established by the Qilu Hospital of Shandong University Committee on Animal Care and Use. At the end of each in vivo experiment, the mice were euthanized by carbon dioxide (CO_2_) inhalation in accordance with animal care guidelines. The Medical Ethics Committee at Qilu Hospital of Shandong University granted approval and oversight for all conducted experiments (DWLL‐2024‐310).

### 2.15 Immunohistochemistry (IHC)

A 4% paraformaldehyde solution was utilized for the fixation of murine tumour tissues. Sections were incubated at 60°C for 1 h, then deparaffinized and rehydrated before antigen retrieval. The samples were incubated with a peroxidase blocking solution at ambient temperature for a duration of 10 min. Following this, blocking was performed for 2 h at 37°C utilizing 5% normal goat serum in PBST. Subsequently, sections were subjected to an overnight incubation at 4°C with the primary antibodies. An HRP‐conjugated secondary antibody was applied to each slide, followed by three washes with TBST, development with DAB solution and counterstaining with haematoxylin. Finally, images of the slides were captured using a microscope, and statistical analysis of the positive cell rate was performed for the staining results.

### 2.16 Statistical Analysis

All quantitative in vitro data are presented as the mean ± standard deviation (SD) from at least three independent biological replicates (*n* = 3). In vivo tumour growth curves are depicted as the mean ± standard error of the mean (SEM). Statistical analyses were performed using GraphPad Prism 9.0 (GraphPad Software, San Diego, CA, USA) and R software (version 4.2.0). Two‐group comparisons were assessed using two‐tailed paired or unpaired Student's *t*‐tests. Multiple‐group comparisons were analysed using one‐way analysis of variance (ANOVA) followed by Tukey's post hoc test. Survival distributions were analysed using the Kaplan–Meier method and compared via log‐rank tests. Correlations between continuous variables were evaluated using Pearson's or Spearman's correlation coefficients. Associations with clinicopathological characteristics were assessed using Fisher's exact test or chi‐square tests. A two‐sided *p*‐value < 0.05 was considered statistically significant. *p < 0.05, **p < 0.01, ***p < 0.001, ****p < 0.0001; ns, not significant.

## 3 Results

To identify novel autophagy‐related prognostic biomarkers in LUAD, we constructed a prognostic risk model using autophagy‐related genes from public databases. A schematic overview of the study design and major findings is presented in Graphical Abstract.

### 3.1 Identification of NLRC4 as a Critical Autophagy‐Related Protective Factor Associated With Favourable Clinical Outcomes Across Multiple LUAD Cohorts

To systematically identify autophagy‐related prognostic biomarkers in lung adenocarcinoma (LUAD), we initially analysed 30 differentially expressed autophagy‐related genes (DEGs) obtained from TCGA‐LUAD and the HADb database (Table S3; Figure S1A–C). Univariate Cox regression analysis screened out 10 survival‐associated candidate genes (Figure S1D), from which multivariate Cox proportional hazards regression established a robust six‐gene prognostic signature comprising NLRC4, BIRC5, P4HB, ITGB4, DLC1 and ERO1A (Figure S1E). The risk score was calculated as: (BIRC5 × 0.125) + (NLRC4 × −0.399) + (P4HB × 0.106) + (ITGB4 × 0.106) + (DLC1 × 0.119) + (ERO1A × 0.292). Notably, NLRC4 exhibited the highest negative coefficient, underscoring its unique role as a predominant protective factor. Patients in the high‐risk group demonstrated significantly poorer overall survival (OS) compared to the low‐risk group (*p* < 0.001, Figure S1F). Time‐dependent ROC curves confirmed the predictive accuracy of the model (Figure S1G). Subsequent univariate Cox regression analysis (Figure S1H) and multivariate Cox analysis (Figure S1I) confirmed grade and risk score as independent prognostic predictors. Risk map analysis showed that low NLRC4 expression correlated with increased risk (Figure S2A–C), and NLRC4 expression was significantly associated with age and clinical stages (Figure S2D–I).

To corroborate the clinical relevance of NLRC4 across broader populations, we performed cross‐database and multi‐cohort analyses. Pan‐cancer evaluation demonstrated that NLRC4 expression was widely suppressed in multiple malignancies, including LUAD (Figure S3A,B). Significant transcriptional downregulation of NLRC4 in LUAD tumours was further verified in TCGA‐LUAD (*p* < 0.001, Figure S3C). Kaplan–Meier survival curves showed that high NLRC4 mRNA levels correlated with prolonged OS in the TCGA cohort (*p* < 0.001, Figure S3D) as well as across four independent GEO validation datasets (GSE50081, GSE31210, GSE11969 and GSE68465; all *p* < 0.05, Figure S3E–H). Clinically, diminished NLRC4 expression significantly correlated with advanced clinical stages and lymph node metastasis (Figure S3I–K). Importantly, subgroup survival analyses indicated that the favourable prognostic value of NLRC4 was robustly maintained regardless of EGFR or KRAS mutation status (Figure S4), demonstrating that its clinical predictive power is independent of major oncogenic driver mutations.

To experimentally validate these bioinformatic findings at the protein level, we examined an institutional cohort of 30 paired LUAD tissues and matched adjacent non‐tumour tissues. In full agreement with the transcriptomic data, immunoblotting of the clinical samples demonstrated marked downregulation of NLRC4 protein in tumour tissues compared to adjacent normal counterparts (Figure 1A). Densitometric quantification confirmed that NLRC4 protein levels were significantly decreased in LUAD tumours (Figure 1B), with paired scatter analysis revealing a consistent decline across matched tissue pairs (Figure 1C). Because NLRC4 was identified as a core protective autophagy‐related factor, and the classical tumour suppressor PTEN serves as the master upstream gatekeeper of autophagy, we simultaneously evaluated PTEN in the exact same patient lysates. Strikingly, paired analysis similarly demonstrated a synchronized, significant reduction of PTEN protein levels across the matched patient tumours (Figure 1D). Furthermore, Kaplan–Meier survival analysis confirmed that patients harbouring low NLRC4 protein expression had significantly shorter OS (*p* < 0.05, Figure 1E). Correlation analysis with clinicopathological parameters revealed that low NLRC4 expression was significantly associated with advanced TNM stage (*p* < 0.0001) and pleural invasion (*p* = 0.031) (Table 1). Taken together, these multidimensional clinical and translational findings establish NLRC4 as an independent autophagy‐associated protective biomarker that is downregulated in LUAD alongside PTEN, prompting our subsequent dissection of its functional and mechanistic tumour‐suppressive role.

**FIGURE 1:**
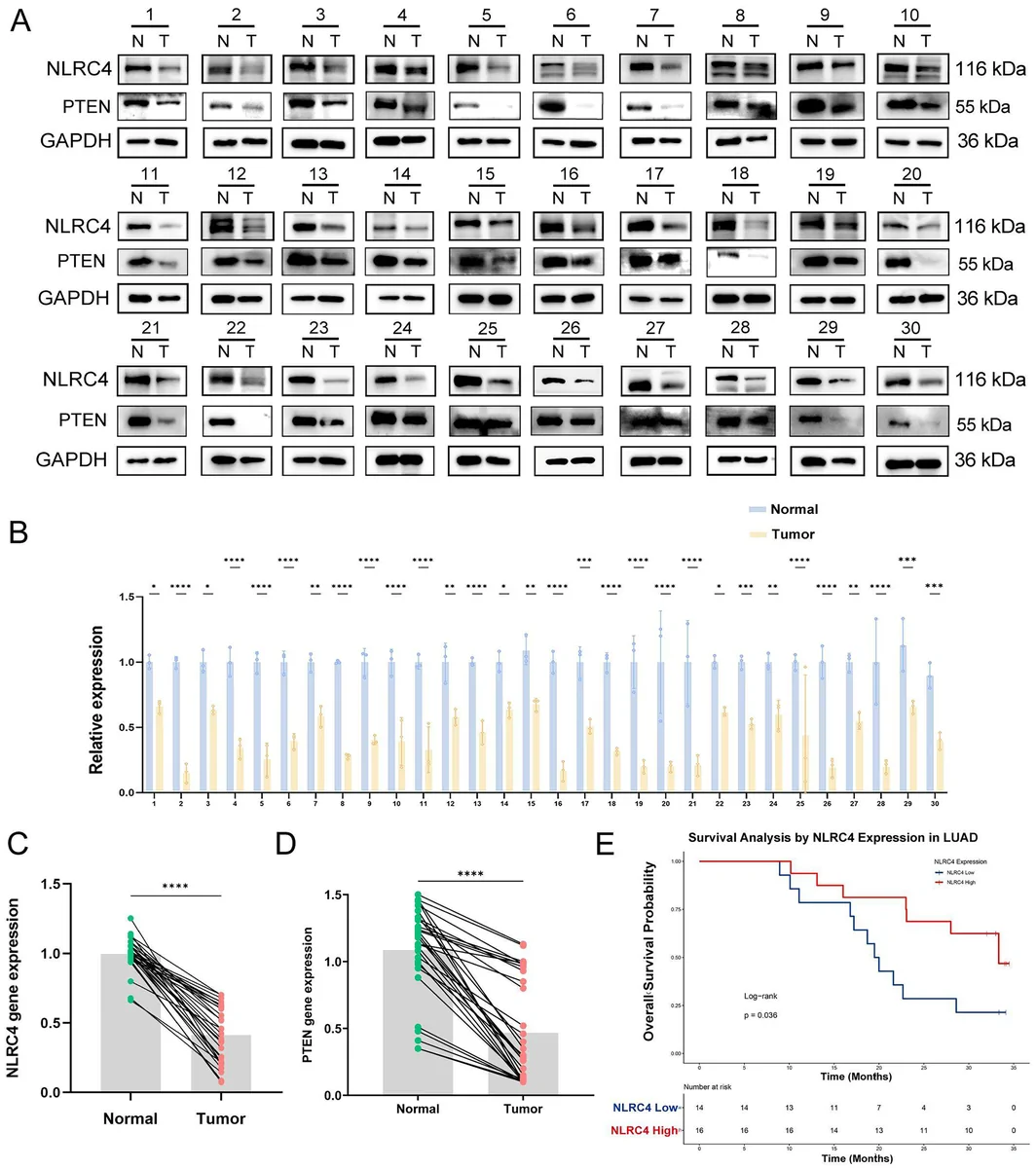
NLRC4 protein is downregulated in LUAD tissues alongside PTEN and correlates with patient survival. (A) Representative Western blot images showing NLRC4 and PTEN protein expression in paired LUAD (T) and adjacent non‐tumour (N) tissues. GAPDH served as the shared loading control for both target proteins sequentially probed on the same set of matched clinical lysates. Patient numbers are indicated above each lane. Representative blots from three independent experiments are shown. (B) Densitometric quantification of relative NLRC4 protein levels normalized to GAPDH in tumour versus adjacent non‐tumour tissues (*n* = 30 pairs). Data are presented as mean ± SD. Statistical significance was determined by paired two‐tailed Student's *t*‐test. (C) Paired scatter plot illustrating NLRC4 protein expression changes in 30 individual matched tumour and non‐tumour tissue pairs. Lines connect matched samples from the same patient. (D) Paired scatter plot illustrating PTEN protein expression changes in the same 30 individual matched tumour and non‐tumour tissue pairs. Lines connect matched samples from the same patient. Statistical significance was determined by paired two‐tailed Student's *t*‐test. (E) Kaplan–Meier survival analysis of overall survival (OS) in the institutional cohort (*n* = 30), stratified by the median NLRC4 protein expression level. The log‐rank test was used to compare survival distributions. CI, confidence interval; HR, hazard ratio. *p < 0.05, **p < 0.01, ***p < 0.001, ****p < 0.0001.

**TABLE 1:** Association between NLRC4 expression levels and clinicopathological characteristics of LUAD patients (*N* = 30).

Characteristics	Numbers	NLRC4 low	NLRC4 high	*p*
Age (years)				
< 60	12	5	7	
≥ 60	18	9	9	0.722
Gender				
Female	19	11	8	
Male	11	3	8	0.142
Tumour size				
≤ 5 cm	25	12	13	
> 5 cm	5	2	3	1.000
Lymphatic metastasis				
Negative	23	9	14	
Positive	7	5	2	0.204
Metastasis				
M0	28	13	15	
M1	2	1	1	1.000
TNM stage				
I–II	18	3	15	
III–IV	12	11	1	< 0.001
Pleural invasion				
+	7	6	1	
−	23	8	15	0.031
STAS				
+	13	9	4	
−	17	5	12	0.063
ALK				
+	5	3	2	
−	25	11	14	0.642
EGFR				
+	10	5	5	
−	20	9	11	1.000

*Note:* Patients were stratified into NLRC4‐low and NLRC4‐high groups based on the median value of NLRC4 protein expression determined by densitometric analysis of Western blots. *p*‐values were calculated using Fisher's exact test. Bold values indicate statistical significance (*p* < 0.05).

Abbreviations: ALK, anaplastic lymphoma kinase; EGFR, epidermal growth factor receptor; LUAD, lung adenocarcinoma; STAS, spread through air spaces; TNM, tumour node metastasis.

### 3.2 Bioinformatic Analyses Reveal a Preferential Association of NLRC4 With Autophagy Over Canonical Inflammasome Activation in LUAD

To globally delineate the downstream signalling pathways and biological functions associated with NLRC4 in LUAD, we first performed comprehensive enrichment analyses using the TCGA‐LUAD cohort. Gene Set Enrichment Analysis (GSEA) revealed a prominent and positive enrichment of core autophagy‐related gene sets in the NLRC4‐high patient subgroup (NES > 1.0, FDR *q* < 0.05; Figure 2A). Consistently, Kyoto Encyclopedia of Genes and Genomes (KEGG) pathway analysis identified the ‘Autophagy—animal’ pathway as significantly enriched (Figure 2B). In full agreement with these findings, Gene Ontology (GO) biological process analysis demonstrated marked over‐representation of terms related to autophagy initiation and intracellular catabolic processes (Figure 2C).

**FIGURE 2:**
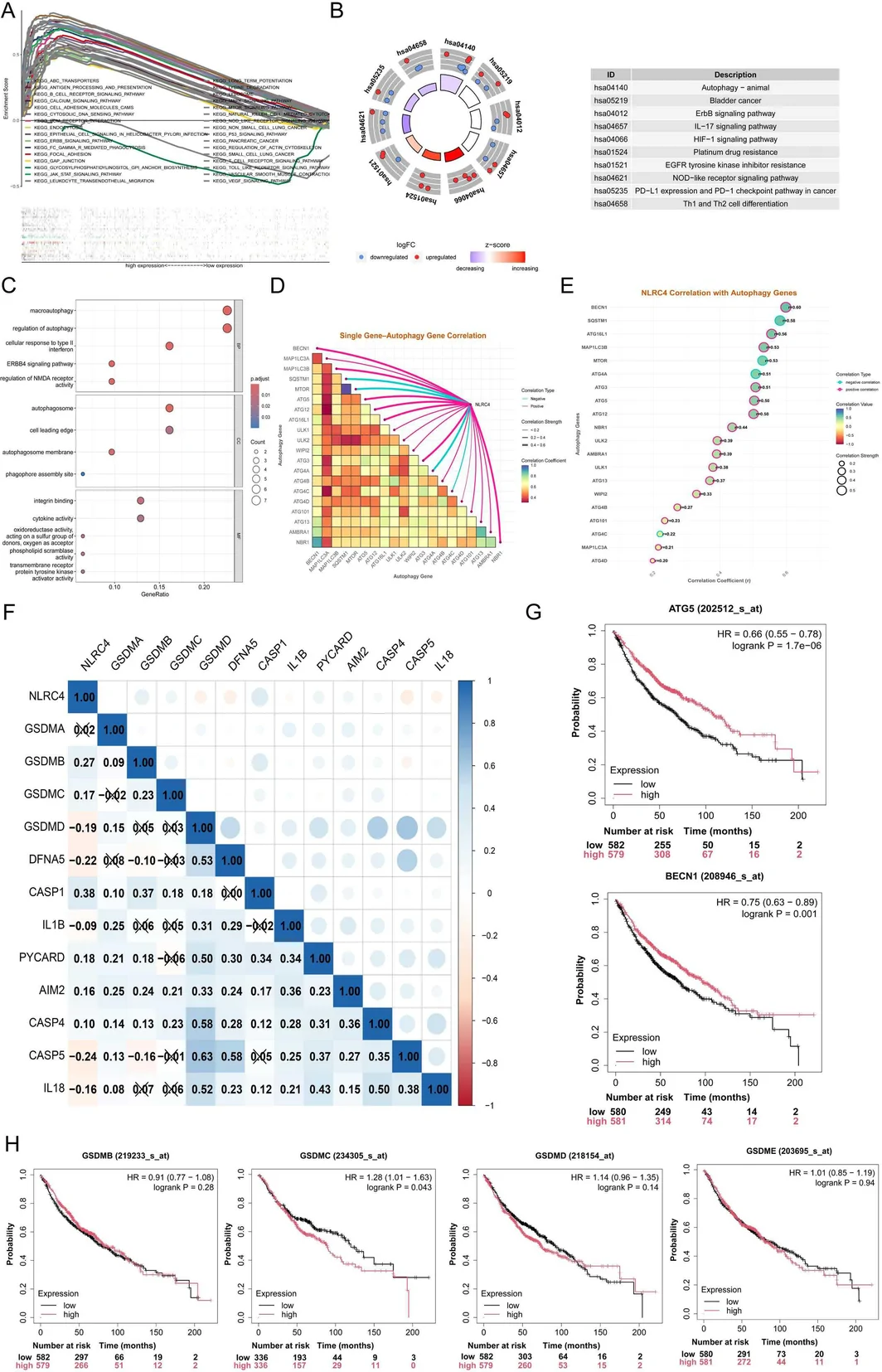
Bioinformatic analyses reveal a preferential association of NLRC4 with autophagy over canonical inflammasome activation and pyroptosis in LUAD. (A) Gene Set Enrichment Analysis (GSEA) plot showing significant enrichment of autophagy‐related gene signatures in the NLRC4‐high TCGA‐LUAD cohort (NES > 1.0, FDR *q*‐value < 0.05). (B) Kyoto Encyclopedia of Genes and Genomes (KEGG) pathway analysis demonstrating significant enrichment of the ‘Autophagy—animal’ pathway. (C) Gene Ontology (GO) enrichment analysis showing over‐represented autophagy‐related biological processes. (D) Heatmap of Spearman correlation coefficients between NLRC4 and core autophagy‐related genes (ARGs) in the TCGA‐LUAD cohort. (E) Representative scatter plots illustrating significant positive correlations between NLRC4 and key ARGs (BECN1 and MAP1LC3B). (F) Heatmap of Spearman correlation coefficients between NLRC4 and canonical inflammasome‐ and pyroptosis‐related genes. (G) Kaplan–Meier survival curves for overall survival (OS) stratified by the median expression of core autophagy genes (ATG5 and BECN1) in TCGA‐LUAD. (H) Kaplan–Meier survival curves for overall survival (OS) stratified by the median expression of Gasdermin family members (GSDMB, GSDMC, GSDMD and GSDME) in TCGA‐LUAD. *p*‐values for survival curves were calculated using the log‐rank test; correlation coefficients (*R*) and *p*‐values were determined by Spearman correlation analysis. *p < 0.05, **p < 0.01, ***p < 0.001, ****p < 0.0001.

To validate this functional link at the individual gene level, we systematically analysed the co‐expression patterns between NLRC4 and autophagy‐related genes (ARGs). Correlation heatmap profiling demonstrated broad and robust positive correlations between NLRC4 and an extensive panel of ARGs in LUAD tissues (Figure 2D). Specifically, scatter plots confirmed strong and statistically significant positive correlations between NLRC4 and key autophagy executioners, including BECN1 and MAP1LC3B (Figure 2E), as well as ATG5 (Figure S5A,B).

Because NLRC4 is classically defined as an inflammasome sensor, we next examined whether its expression is functionally coupled to canonical inflammasome components or pyroptotic executioners in LUAD. Correlation heatmap analysis revealed that NLRC4 exhibited notably weaker or disjointed correlations with inflammasome‐ and pyroptosis‐associated genes (Figure 2F). In‐depth scatter plot analyses further demonstrated that NLRC4 had weak or non‐significant correlations with Gasdermin family executioners (GSDMB, GSDMC, GSDMD and GSDME) (Figure S5C–F), and showed a comparatively weak correlation with the indispensable inflammasome adaptor PYCARD/ASC (*R* = 0.372) than with other inflammasome components such as IL1B and CASP1 (Figure S5G), suggesting that NLRC4 is largely uncoupled from canonical inflammasome assembly in LUAD.

Finally, to address whether patient survival in LUAD is governed by autophagic or pyroptotic machinery, we compared the prognostic value of these two functional gene sets. Kaplan–Meier survival analyses demonstrated that high expression of core autophagy drivers (ATG5 and BECN1) was significantly associated with favourable overall survival (*p* < 0.05; Figure 2G), strictly mirroring the protective phenotype of NLRC4. In sharp contrast, expression levels of Gasdermin family members (GSDMB–GSDME) failed to confer significant survival advantages or exhibited inverse prognostic correlations (Figure 2H). Collectively, these transcriptomic and clinical profiling data establish that NLRC4 is preferentially wired to the autophagic machinery rather than canonical inflammasome and pyroptotic pathways in LUAD.

### 3.3 NLRC4 Activates Autophagy and Enhances Autophagic Flux Independently of Canonical Pyroptosis in LUAD Cells

Mining of the CCLE and HPA databases confirmed that baseline NLRC4 expression was markedly suppressed in human LUAD cell lines and patient tumours (Figure S6A,B). Consistently, baseline evaluation via RT‐qPCR and Western blot revealed lower endogenous NLRC4 levels across four LUAD lines (A549, H1975, PC9, H1299) compared with normal bronchial epithelial BEAS‐2B cells (Figure S6C,D). We then established stable NLRC4 overexpression (OE) and siRNA knockdown (siNLRC4) models across lines with distinct oncogenic backgrounds, including A549 (KRAS‐mutant), H1975 (EGFR‐mutant) and H1299 (TP53‐null) (Figure S6E,F).

Western blot analysis demonstrated that NLRC4 overexpression upregulated Beclin‐1, increased the LC3B‐II/I ratio, and promoted p62 degradation, whereas NLRC4 silencing caused opposite changes (Figure 3A,B). Transmission electron microscopy (TEM) confirmed a higher density of autophagic vacuoles (both autophagosomes and autolysosomes) in NLRC4‐OE cells (Figure 3C,D). To determine whether this accumulation reflected enhanced autophagic activity rather than impaired degradation, cells were transduced with the mRFP‐GFP‐LC3B tandem reporter. NLRC4 overexpression predominantly increased red‐only puncta (GFP^−^/RFP^+^, autolysosomes) rather than yellow puncta (GFP^+^/RFP^+^, autophagosomes) (Figure 3E,F), indicating functional autophagosome‐lysosome fusion and completed autophagic flux. In parallel, blocking lysosomal degradation with Bafilomycin A1 (Baf A1) resulted in a significantly higher net accumulation of LC3B‐II (ΔLC3B‐II) in NLRC4‐OE cells compared to controls (Figure 3G–I), confirming that NLRC4 genuinely drives complete autophagic flux.

**FIGURE 3:**
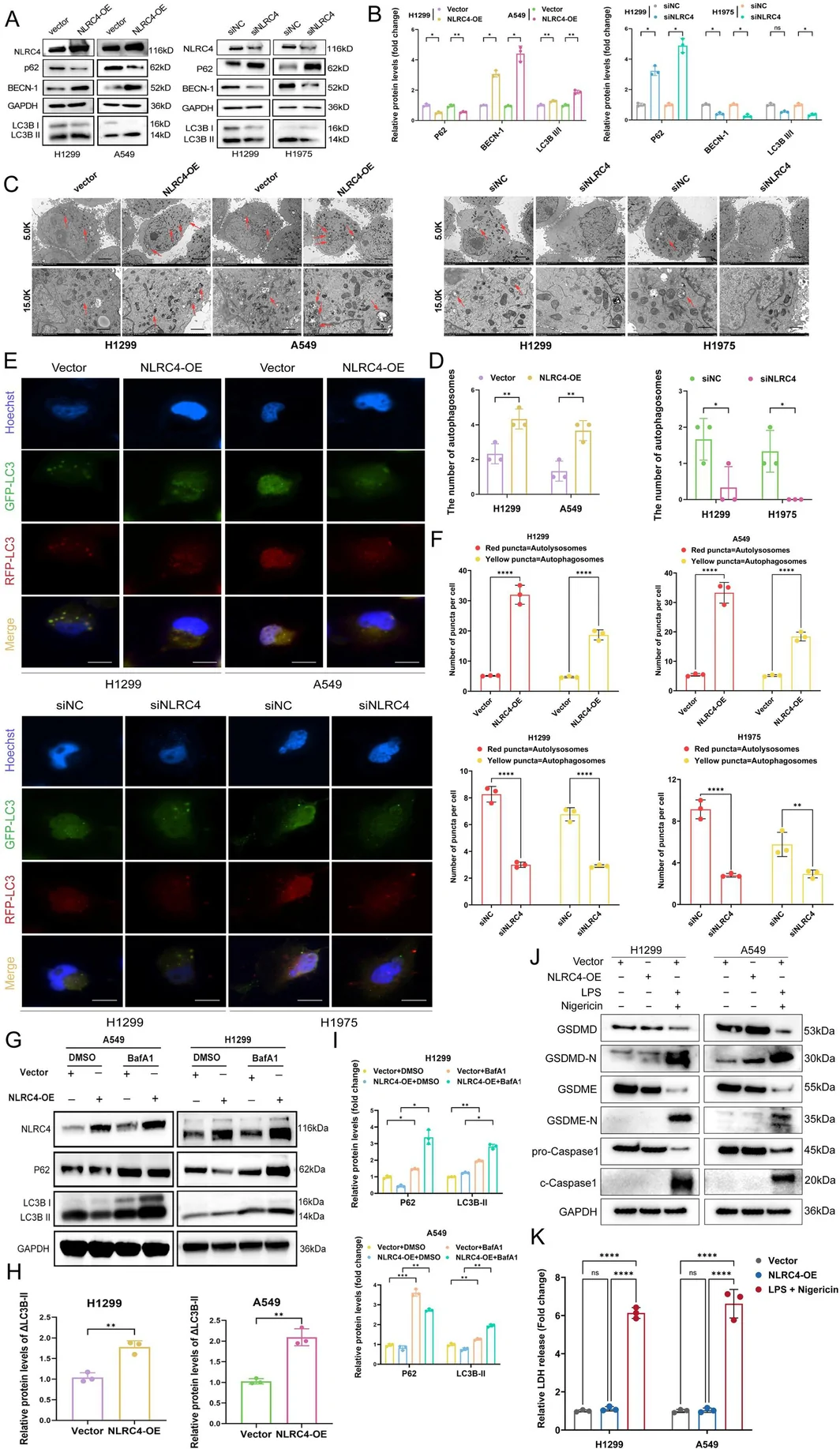
NLRC4 activates autophagy and enhances autophagic flux independently of canonical pyroptosis in LUAD cells. (A) Western blot analysis of autophagy markers (NLRC4, p62, LC3B‐I/II and Beclin‐1) in H1299, A549 and H1975 cells following NLRC4 overexpression (NLRC4‐OE) or knockdown (siNLRC4). (B) Densitometric quantification of p62, LC3B‐II/I and Beclin‐1 levels from (A). (C) Representative transmission electron microscopy (TEM) images of autophagic ultrastructures. Arrows indicate typical autophagic vacuoles (autophagosomes and autolysosomes). Scale bars: 2 μm (upper panels) and 0.5 μm (lower magnified views). (D) Morphometric quantification of autophagic vacuole numbers per cell from TEM sections (*n* ≥ 20 cells/group). (E) Confocal fluorescence images of cells stably expressing the mRFP‐GFP‐LC3B reporter. Merged yellow puncta (GFP^+^/RFP^+^) indicate autophagosomes; red‐only puncta (GFP^−^/RFP^+^) reflect autolysosomes due to lysosomal quenching of GFP. Scale bar: 10 μm. (F) Quantification of autophagosomes (yellow puncta) and autolysosomes (red‐only puncta) per cell (*n* ≥ 30 cells/group). (G) Autophagic flux assessment by Western blot detection of LC3B‐II and p62 in cells treated with vehicle or Bafilomycin A1 (Baf A1; 100 nM, 4 h). (H) Autophagic flux rate calculated as ΔLC3B‐II accumulation. (I) Densitometric analysis of p62 and LC3B‐II levels from (G). (J) Western blot analysis of Caspase‐1 (pro‐form and active p20), GSDMD (full‐length and cleaved‐N) and GSDME (full‐length and cleaved) in H1299 and A549 cells. Cells treated with LPS (1 μg/mL, 4 h) plus Nigericin (10 μM, 1 h) served as a technical positive control. (K) Extracellular LDH release cytotoxicity assay. GAPDH was used as the loading control for Western blots. Data are presented as mean ± SD from three independent biological replicates (*n* = 3). Statistical comparisons were evaluated by unpaired two‐tailed Student's *t*‐test or one‐way ANOVA with Tukey's post hoc test. *p < 0.05, **p < 0.01, ***p < 0.001, ****p < 0.0001; ns, not significant.

Although NLRC4 is classically defined as an inflammasome sensor, our bioinformatic analyses suggested its decoupling from canonical pyroptosis in LUAD. To experimentally confirm this in vitro, we tested whether ectopic NLRC4 triggers pyroptotic cell death in LUAD cells. We used LPS plus Nigericin treatment as a technical positive control to demonstrate that the downstream execution machinery remained responsive in these cells. While the positive control induced robust cleavage of Caspase‐1 (active p20 form) and Gasdermins (cleaved GSDMD and GSDME) (Figure 3J) along with extensive LDH release (Figure 3K), NLRC4 overexpression failed to trigger detectable Caspase‐1/Gasdermin cleavage or increase extracellular LDH levels (Figure 3J,K).

Together, these data provide direct biochemical and functional evidence that NLRC4 selectively stimulates complete autophagic flux without activating canonical pyroptosis in LUAD cells.

### 3.4 The Tumour‐Suppressive Function of NLRC4 Is Dependent on Autophagy Machinery in LUAD Cells

We next evaluated the functional impact of NLRC4 on LUAD malignant phenotypes in vitro. CCK‐8 proliferation curves showed that NLRC4 overexpression significantly impeded cell growth from 48 to 96 h, whereas NLRC4 silencing promoted cell proliferation (Figure 4A,B). Consistently, EdU incorporation assays demonstrated a marked reduction in DNA synthesis following NLRC4 overexpression, while knockdown yielded the opposite effect (Figure 4C,D). In parallel, wound healing and Transwell assays revealed that NLRC4 overexpression robustly constrained motility and Matrigel invasion, whereas NLRC4 depletion markedly accelerated both migratory and invasive capabilities (Figure 4E–H). Furthermore, immunoblotting showed that NLRC4 overexpression upregulated the epithelial marker E‐cadherin while repressing mesenchymal markers (N‐cadherin, Vimentin and Snail), indicating the suppression of epithelial–mesenchymal transition (EMT) (Figure 4I,J).

**FIGURE 4:**
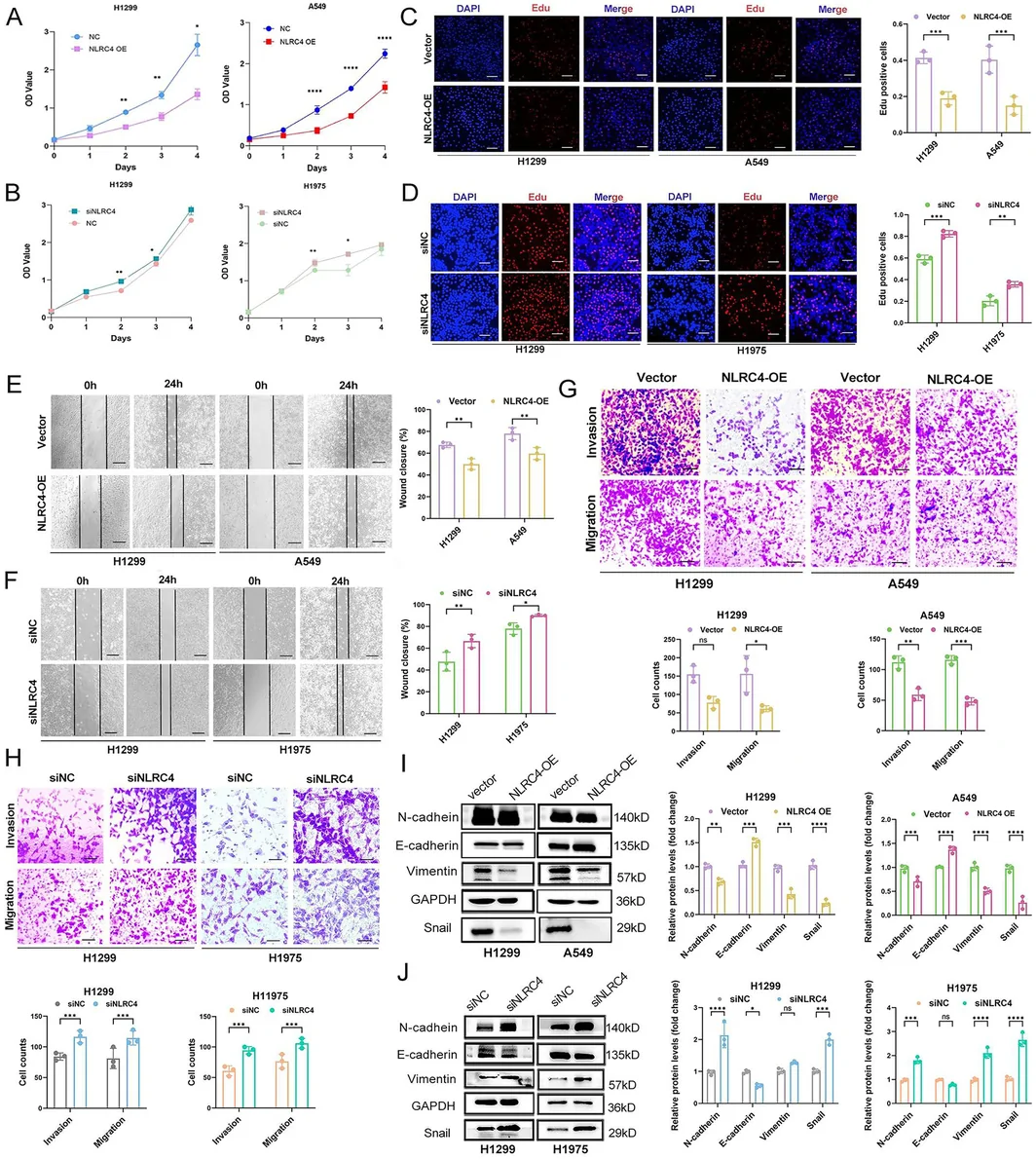
NLRC4 suppresses proliferation, migration, invasion and EMT in LUAD cells. (A, B) Cell proliferation assessed by CCK‐8 assays at indicated time points (0–96 h) in NLRC4‐overexpressing (A) and NLRC4‐knockdown (B) LUAD cells. (C, D) Representative micrographs (left) and quantification (right) of EdU incorporation assays in NLRC4‐overexpressing (C) and NLRC4‐knockdown (D) cells. Red, EdU^+^; blue, Hoechst 33342. Scale bar: 100 μm. (E, F) Wound‐healing assays evaluating cell migration in NLRC4‐overexpressing (H1299, A549) (E) and NLRC4‐knockdown (H1299, H1975) (F) cells at 0 and 24 h post‐scratch. Dashed lines mark the wound edges. Scale bar: 200 μm. (G, H) Transwell migration and Matrigel invasion assays in NLRC4‐overexpressing (G) and NLRC4‐knockdown (H) cells after 24 h incubation. Scale bar: 100 μm. (I, J) Western blot analysis (left) and densitometric quantification (right) of EMT markers (E‐cadherin, N‐cadherin, Vimentin and Snail) in NLRC4‐overexpressing (I) and NLRC4‐knockdown (J) cells. GAPDH was used as the loading control for Western blots. Data are presented as mean ± SD from three independent biological replicates (*n* = 3). Statistical comparisons were evaluated by unpaired two‐tailed Student's *t*‐test or one‐way ANOVA with Tukey's post hoc test. *p < 0.05, **p < 0.01, ***p < 0.001, ****p < 0.0001.

To establish whether this tumour‐suppressive activity is causally linked to autophagic activation, we performed bidirectional pharmacological rescue experiments. In NLRC4‐overexpressing cells, chloroquine (CQ) treatment effectively blocked autophagic degradation (Figure 5A,B) and caused an accumulation of yellow autophagosome puncta (Figure 5C,D). Crucially, CQ‐mediated autophagy blockade substantially restored cellular proliferation, migration and Matrigel invasion in NLRC4‐overexpressing cells (Figure 5E–J). Conversely, in NLRC4‐knockdown cells where autophagic flux was suppressed, pharmacological induction of autophagy via rapamycin (Figure 6A–D) effectively rescued the accelerated proliferation, migratory and invasive phenotypes triggered by NLRC4 loss (Figure 6E–J).

**FIGURE 5:**
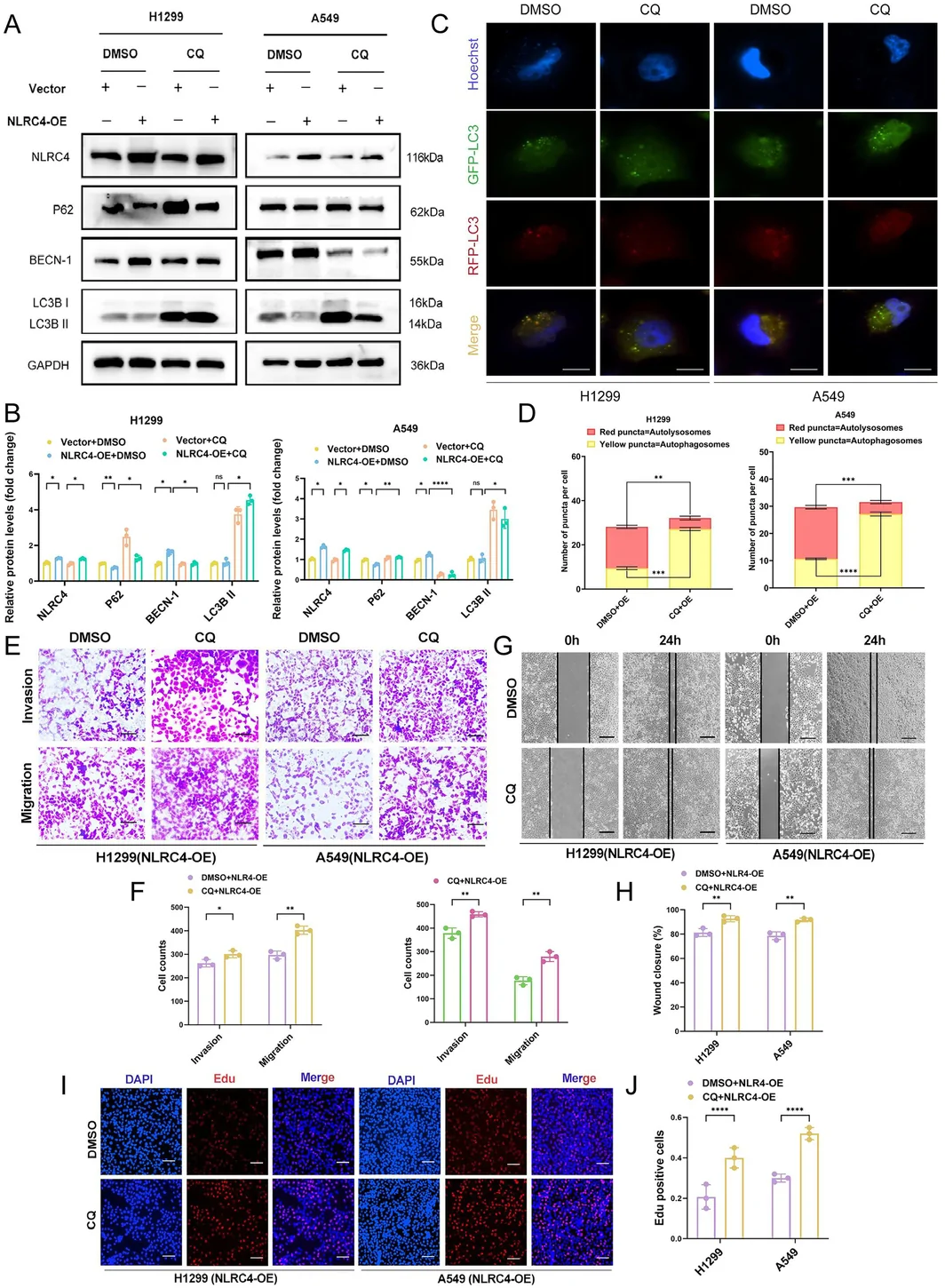
Chloroquine (CQ)‐mediated autophagy inhibition reverses the tumour‐suppressive effects of NLRC4 overexpression in LUAD cells. (A) Western blot analysis of autophagy markers (p62, LC3B‐I/II and Beclin‐1) in NLRC4‐overexpressing or control (Vector) H1299 and A549 cells treated with vehicle (DMSO) or chloroquine (CQ; 20 μM, 24 h). (B) Densitometric quantification of p62, LC3B‐II/I and Beclin‐1 levels from (A). (C) Representative confocal fluorescence images of cells stably expressing the mRFP‐GFP‐LC3B reporter across the indicated treatment groups. Scale bar: 10 μm. (D) Quantification of autophagic flux calculated as the ratio of red‐only (GFP^−^/RFP^+^) to total puncta (*n* ≥ 30 cells/group). (E) Representative images of Transwell migration and Matrigel invasion assays in the indicated treatment groups. Scale bar: 100 μm. (F) Quantitative analysis of migrated and invaded cells from (E). (G) Wound‐healing scratch assays evaluated at 0 and 24 h post‐scratch. Dashed lines delineate the wound margins. Scale bar: 200 μm. (H) Quantification of wound closure rate from (G). (I) Representative fluorescence micrographs of EdU incorporation assays. Scale bar: 100 μm. (J) Quantification of EdU‐positive proliferating cells from (I). Data are presented as mean ± SD from three independent biological replicates (*n* = 3). Statistical comparisons were evaluated by one‐way ANOVA with Tukey's post hoc test. *p < 0.05, **p < 0.01, ***p < 0.001, ****p < 0.0001; ns, not significant.

**FIGURE 6:**
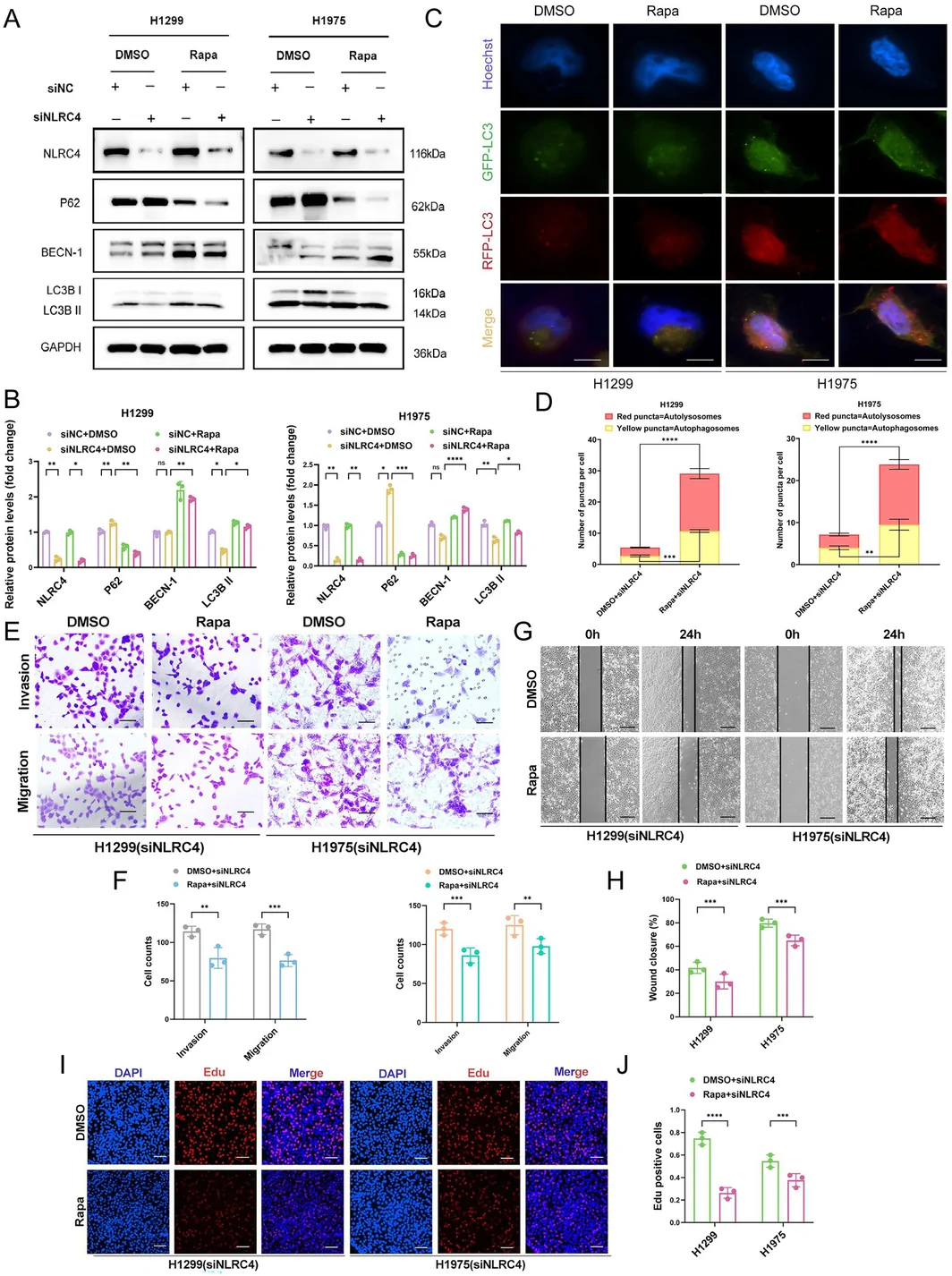
Rapamycin (Rapa)‐mediated autophagy induction reverses the oncogenic phenotypes of NLRC4 knockdown in LUAD cells. (A) Western blot analysis of autophagy markers (p62, LC3B‐I/II and Beclin‐1) in NLRC4‐knockdown or control (siNC) H1299 and H1975 cells treated with vehicle (DMSO) or rapamycin (Rapa; 100 nM, 24 h). (B) Densitometric quantification of p62, LC3B‐II/I and Beclin‐1 levels from (A). (C) Representative confocal fluorescence images of cells stably expressing the mRFP‐GFP‐LC3B reporter across the indicated treatment groups. Scale bar: 10 μm. (D) Quantification of autophagic flux calculated as the ratio of red‐only (GFP^−^/RFP^+^) to total puncta (*n* ≥ 30 cells/group). (E) Representative images of Transwell migration and Matrigel invasion assays in the indicated treatment groups. Scale bar: 100 μm. (F) Quantitative analysis of migrated and invaded cells from (E). (G) Wound‐healing scratch assays evaluated at 0 and 24 h post‐scratch. Dashed lines delineate the wound margins. Scale bar: 200 μm. (H) Quantification of wound closure rate from (G). (I) Representative fluorescence micrographs of EdU incorporation assays. Scale bar: 100 μm. (J) Quantification of EdU‐positive proliferating cells from (I). GAPDH was used as the loading control for Western blots. Data are presented as mean ± SD from three independent biological replicates (*n* = 3). Statistical comparisons were evaluated by one‐way ANOVA with Tukey's post hoc test. *p < 0.05, **p < 0.01, ***p < 0.001, ****p < 0.0001; ns, not significant.

To corroborate these pharmacological findings with genetic evidence, we silenced ATG5 in NLRC4‐overexpressing cells. Genetic inhibition of autophagy by siATG5 successfully abolished the NLRC4‐induced accumulation of LC3B‐II and degradation of p62 (Figure S7A,B), which completely abrogated the anti‐proliferative advantage conferred by NLRC4 overexpression (Figure S7C,D).

Together, these complementary pharmacological and genetic rescue experiments provide definitive evidence that the tumour‐suppressive role of NLRC4 in LUAD is predominantly dependent on intact autophagic machinery.

### 3.5 NLRC4 Activates Autophagy by Inhibiting the PI3K/AKT/mTOR Signalling Pathway

Having confirmed that NLRC4 suppresses LUAD progression via autophagy, we next explored the upstream cascade driving this response. Because the PI3K/AKT/mTOR axis acts as a primary negative regulator of autophagy, we evaluated whether NLRC4 promotes autophagy by repressing this central signalling cascade.

Western blot analysis demonstrated that NLRC4 overexpression in LUAD cells significantly decreased the phosphorylation levels of PI3K (p‐PI3K) and AKT (p‐AKT), as well as total mTOR expression, without altering total PI3K or AKT protein levels (Figure 7A,B). Conversely, NLRC4 knockdown markedly elevated p‐PI3K, p‐AKT and total mTOR levels (Figure 7A,B), establishing an inverse relationship between NLRC4 expression and PI3K/AKT/mTOR signalling activity.

**FIGURE 7:**
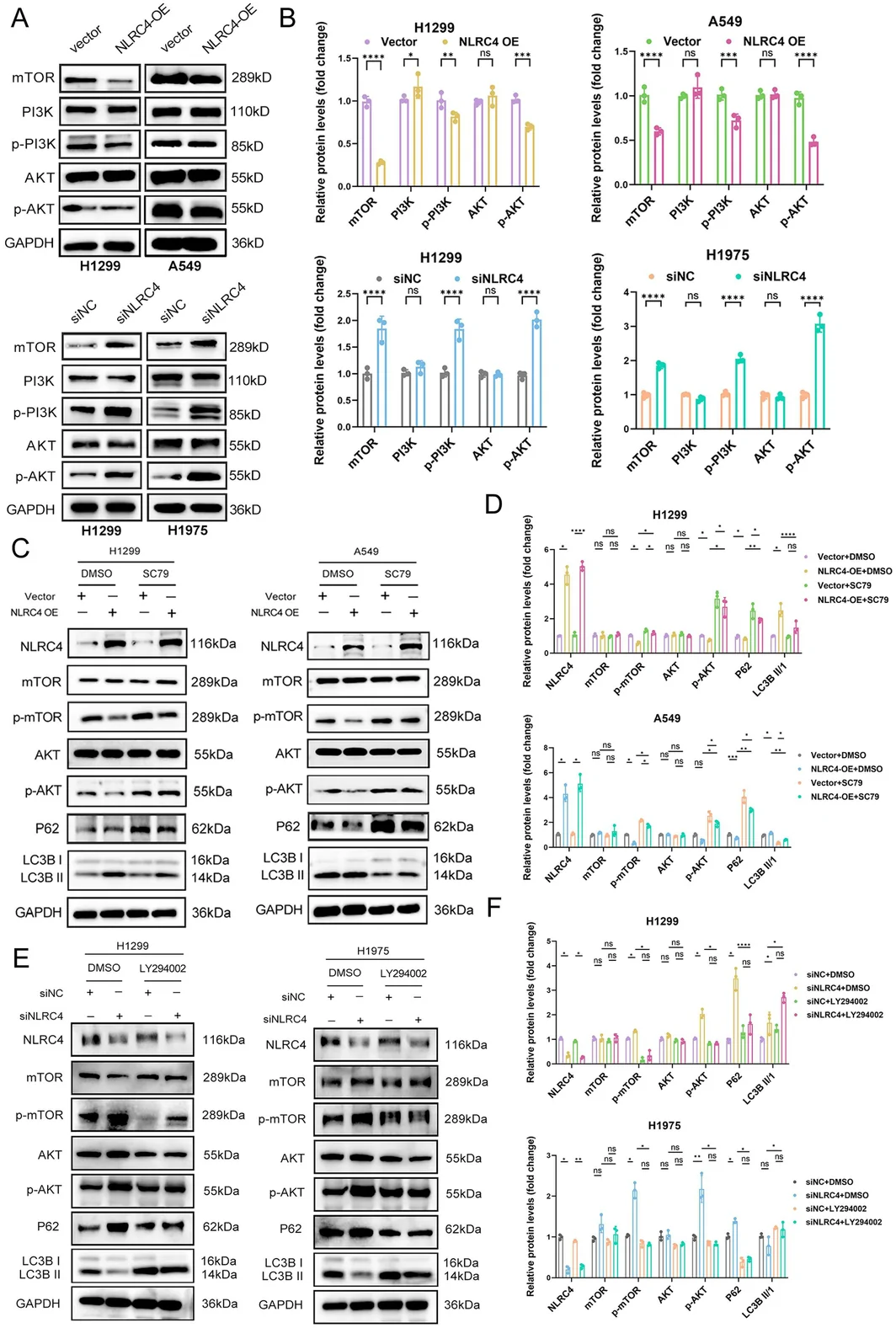
NLRC4 suppresses the PI3K/AKT/mTOR signalling pathway to induce autophagy in LUAD cells. (A) Western blot analysis of PI3K/AKT/mTOR pathway components (p‐PI3K, PI3K, p‐AKT, AKT and mTOR) in H1299, A549 and H1975 cells following NLRC4 overexpression (NLRC4‐OE) or siRNA‐mediated knockdown (siNLRC4). (B) Densitometric quantification of the immunoblots from (A). (C) Western blot analysis evaluating the effect of the AKT activator SC79 (10 μM, 6 h) on pathway reactivation (p‐AKT, AKT, p‐mTOR, mTOR) and autophagy markers (LC3B‐I/II, p62) in NLRC4‐overexpressing H1299 and A549 cells. (D) Densitometric quantification of the immunoblots from (C). (E) Western blot analysis evaluating the effect of the PI3K inhibitor LY294002 (20 μM, 6 h) on pathway suppression and autophagy markers in NLRC4‐knockdown H1299 and H1975 cells. (F) Densitometric quantification of the immunoblots from (E). GAPDH was used as the loading control for Western blots. Data are presented as mean ± SD from three independent biological replicates (*n* = 3). Statistical comparisons were evaluated by one‐way ANOVA with Tukey's post hoc test. *p < 0.05, **p < 0.01, ***p < 0.001, ****p < 0.0001; ns, not significant.

To determine whether pathway suppression is causally required for NLRC4‐driven autophagy, we performed reciprocal pharmacological rescue experiments. In NLRC4‐overexpressing cells, reactivating the pathway with the AKT agonist SC79 restored the phosphorylation of both AKT and mTOR (Figure 7C,D). Concurrently, SC79 treatment reversed NLRC4‐induced autophagic activation, as evidenced by a reduced LC3B‐II/I ratio and the re‐accumulation of p62 (Figure 7C,D).

In a complementary approach, we treated NLRC4‐depleted cells with the PI3K inhibitor LY294002 to counteract the hyperactivated pathway. Administration of LY294002 effectively attenuated the elevated levels of p‐AKT and p‐mTOR, and successfully restored autophagic flux, marked by increased LC3B‐II conversion and enhanced p62 clearance (Figure 7E,F).

Collectively, these bidirectional pharmacological interventions demonstrate that suppression of the PI3K/AKT/mTOR pathway is functionally required for NLRC4‐mediated autophagy activation in LUAD cells.

### 3.6 NLRC4 Interacts With NFKB1, Correlating With PTEN Restoration and PI3K/AKT Pathway Suppression in LUAD

Having established that NLRC4 represses the PI3K/AKT/mTOR axis to trigger protective autophagy, we next investigated the direct molecular mechanism linking this cytoplasmic sensor to upstream pathway suppression. Because NLRC4 lacks intrinsic kinase or phosphatase activity, we hypothesized that it might modulate signalling through novel protein–protein interactions. Interrogation of the STRING database predicted a high‐confidence physical interaction between NLRC4 and NFKB1 (confidence score: 0.72) (Figure 8A), a core transcription factor component of the NF‐κB pathway. To validate this prediction, we performed exogenous co‐immunoprecipitation (Co‐IP) in HEK‐293 T cells co‐transfected with Flag‐tagged NLRC4 and HA‐tagged NFKB1 plasmids, which confirmed a direct physical association (Figure 8B).

**FIGURE 8:**
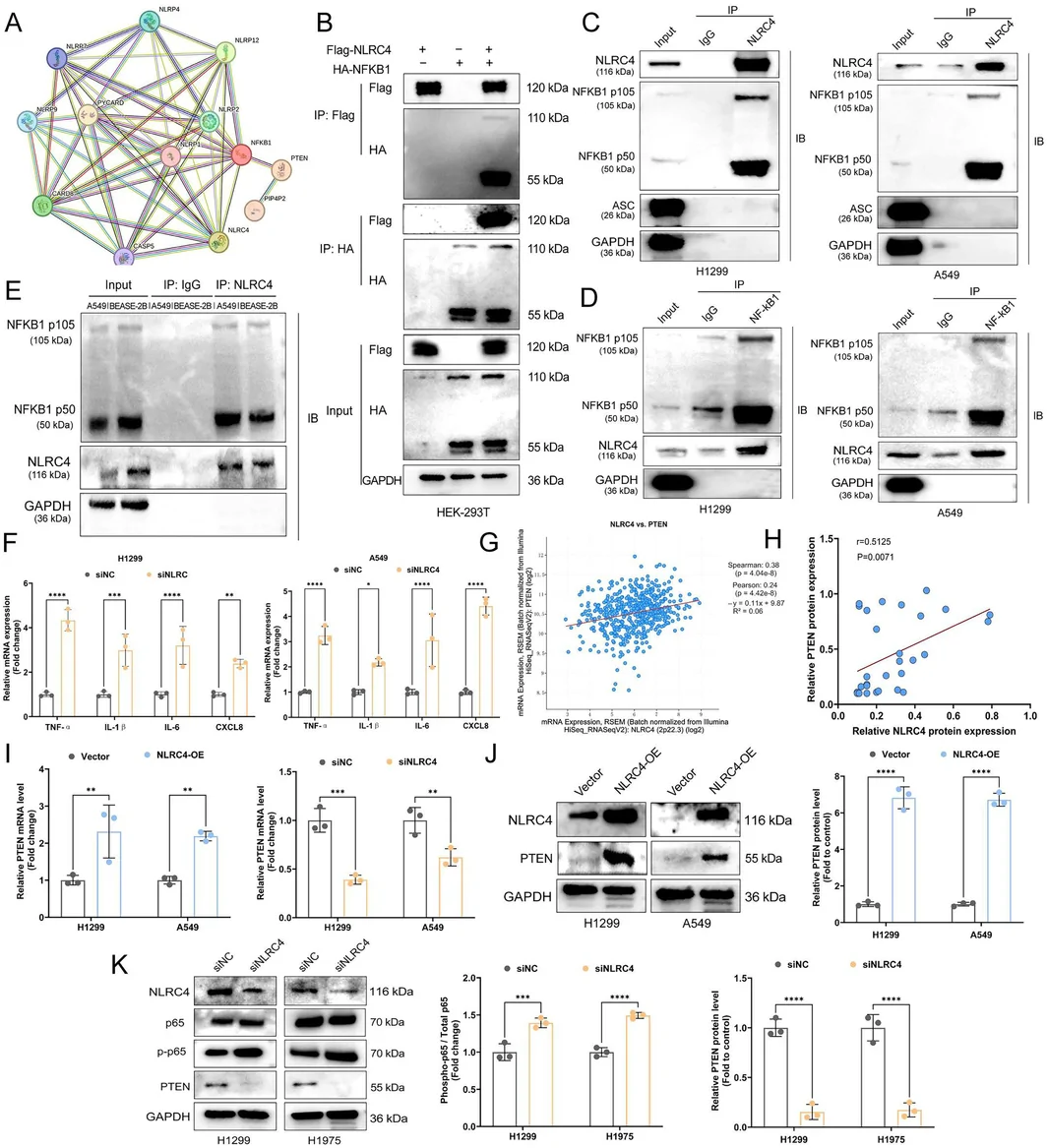
NLRC4 interacts with NFKB1, correlating with PTEN restoration and PI3K/AKT pathway suppression in LUAD. (A) Protein–protein interaction (PPI) network from the STRING database predicting a direct physical interaction between NLRC4 and NFKB1 (confidence score: 0.72). (B) Exogenous co‐immunoprecipitation (Co‐IP) in HEK‐293T cells co‐transfected with Flag‐tagged NLRC4 and HA‐tagged NFKB1 plasmids to detect physical binding. (C) Endogenous Co‐IP in H1299 and A549 cells using an anti‐NLRC4 antibody, with species‐matched IgG as negative control and GAPDH/ASC as specificity controls. (D) Reciprocal endogenous Co‐IP in H1299 and A549 cells using an anti‐NFKB1 antibody, with IgG and GAPDH as controls. (E) Comparative Co‐IP of endogenous NLRC4–NFKB1 interaction between normal bronchial epithelial cells (BEAS‐2B) and LUAD cells (A549). (F) RT‐qPCR analysis of NF‐κB downstream inflammatory target genes (TNF, IL1B, IL6 and CXCL8) in LUAD cells following NLRC4 knockdown. (G) Correlation between NLRC4 and PTEN mRNA expression in LUAD samples from the cBioPortal database. (H) Pearson correlation scatter plot of relative NLRC4 and PTEN protein expression in the institutional cohort of 30 paired LUAD patient tissues. (I) RT‐qPCR analysis of PTEN mRNA expression in LUAD cells following NLRC4 overexpression (OE) or knockdown (siNLRC4). (J) Western blot analysis of PTEN protein expression in NLRC4‐overexpressing LUAD cells (left) and densitometric quantification normalized to GAPDH (right). (K) Western blot analysis of PTEN, p‐p65 and total p65 in NLRC4‐knockdown LUAD cells (left) alongside densitometric quantification (right). Data are presented as mean ± SD from three independent biological replicates (*n* = 3). Statistical comparisons were evaluated by two‐tailed Student's *t*‐test or one‐way ANOVA with Tukey's post hoc test. *p < 0.05, **p < 0.01, ***p < 0.001, ****p < 0.0001.

To ascertain whether this interaction occurs endogenously under physiological conditions, reciprocal Co‐IP assays were conducted in H1299 and A549 LUAD cells. Immunoprecipitation with an anti‐NLRC4 antibody specifically pulled down endogenous NFKB1, while the control IgG, GAPDH and the classical inflammasome adaptor ASC showed no detectable binding (Figure 8C). Conversely, immunoprecipitation with an anti‐NFKB1 antibody efficiently co‐precipitated endogenous NLRC4 (Figure 8D). Furthermore, comparative Co‐IP assays revealed that this NLRC4‐NFKB1 interaction was markedly enriched in malignant LUAD cells (A549) relative to normal bronchial epithelial cells (BEAS‐2B) (Figure 8E), demonstrating a tumour context‐dependent molecular association.

Because the NFKB1 precursor p105 and mature p50 subunits form functional complexes with p65 (RelA) [17] to govern NF‐κB transcriptional outputs, we tested whether NLRC4 depletion unleashes NF‐κB signalling. RT‐qPCR analysis demonstrated that silencing NLRC4 significantly upregulated classical NF‐κB downstream inflammatory target genes (TNF, IL1B, IL6 and CXCL8) (Figure 8F). Given that hyperactivated NF‐κB (specifically p65) acts as a well‐documented transcriptional repressor of the tumour suppressor PTEN [18], we hypothesized that NLRC4‐mediated sequestration of NFKB1 prevents NF‐κB‐dependent PTEN suppression. In support of this model, analysis of the cBioPortal database revealed a positive correlation between NLRC4 and PTEN mRNA expression in LUAD (Figure 8G). Importantly, in our institutional cohort of 30 paired patient tissues, NLRC4 and PTEN protein levels exhibited a highly significant positive correlation (*p* < 0.001, Figure 8H). In vitro, RT‐qPCR confirmed that PTEN mRNA was upregulated by NLRC4 overexpression and downregulated upon NLRC4 knockdown (Figure 8I). At the protein level, NLRC4 overexpression robustly enhanced PTEN expression (Figure 8J). Conversely, NLRC4 depletion simultaneously induced p65 hyperactivation (marked accumulation of p‐p65) and abolished PTEN protein levels (Figure 8K), supporting a signalling cascade wherein NLRC4 restrains NF‐κB activation to restore PTEN expression.

To functionally evaluate whether PTEN is the indispensable mediator of NLRC4‐driven autophagy and tumour suppression, we performed bidirectional genetic rescue experiments (Figure 9). Immunoblotting showed that in NLRC4‐overexpressing cells, silencing PTEN (NLRC4‐OE + siPTEN) effectively reversed AKT dephosphorylation and blocked autophagic induction, as indicated by p62 re‐accumulation and reduced LC3B‐II levels (Figure 9A,B). Restoring PTEN in NLRC4‐knockdown cells (siNLRC4 + PTEN‐OE) substantially attenuated the hyperphosphorylation of AKT and rescued autophagic flux (Figure 9C,D).

**FIGURE 9:**
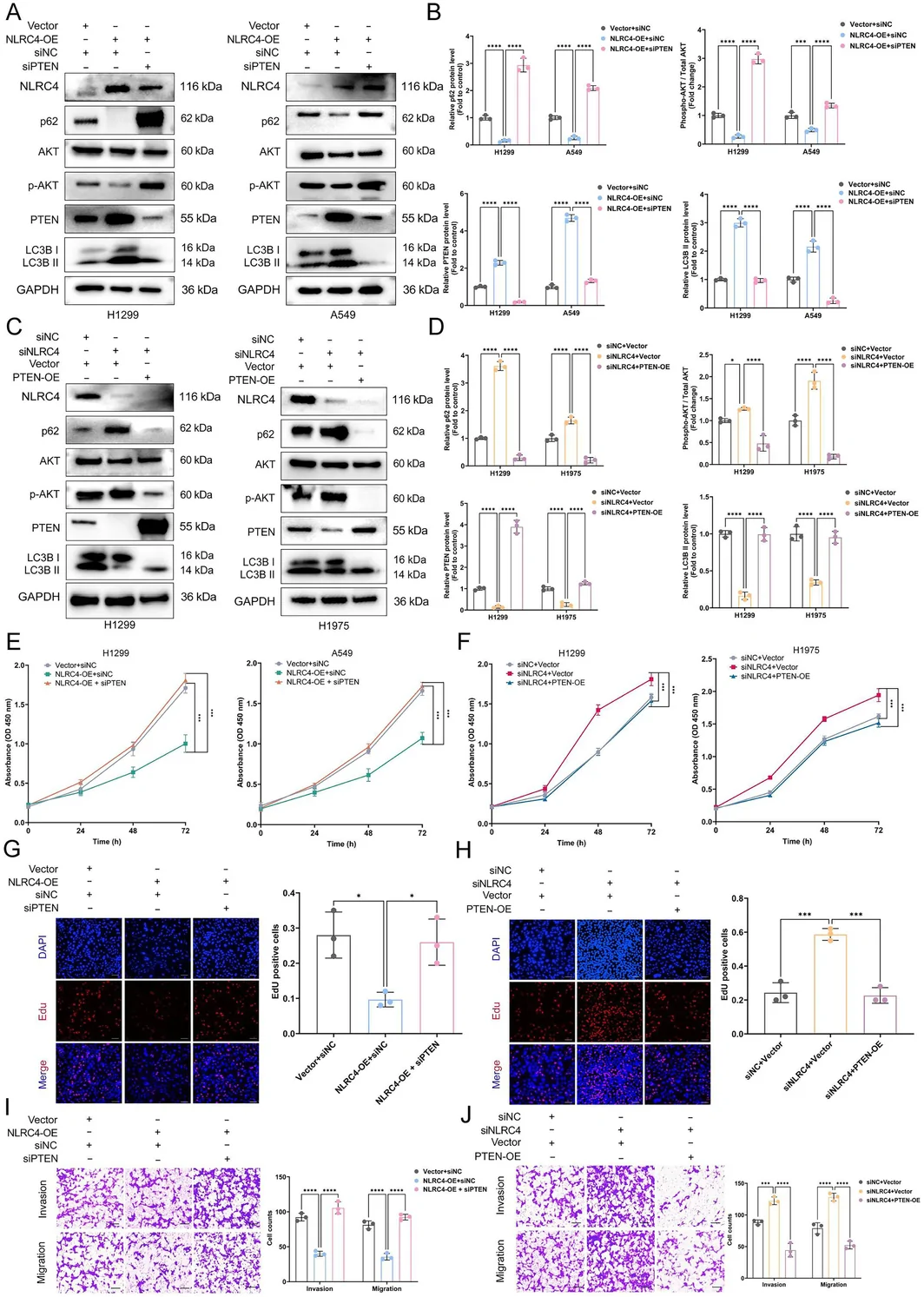
PTEN is functionally required for NLRC4‐mediated autophagy activation and tumour suppression in LUAD cells. (A) Western blot analysis of PTEN, p‐AKT, AKT, LC3B‐I/II and p62 in H1299 and A549 cells transfected with Vector + siNC, NLRC4‐OE + siNC or NLRC4‐OE + siPTEN. (B) Densitometric quantification of the immunoblots from (A). (C) Western blot analysis of PTEN, p‐AKT, AKT, LC3B‐I/II and p62 in H1299 and H1975 cells transfected with siNC + Vector, siNLRC4 + Vector or siNLRC4 + PTEN‐OE. (D) Densitometric quantification of the immunoblots from (C). (E) Cell proliferation assessed by CCK‐8 assay at indicated time points (0–72 h) in A549 and H1299 cells across the indicated transfection groups as in (A). (F) Cell proliferation assessed by CCK‐8 assay at indicated time points (0–72 h) in H1299 and H1975 cells across the indicated transfection groups as in (C). (G) Representative fluorescence micrographs and quantification of EdU incorporation assays in H1299 cells across the indicated transfection groups as in (A). Scale bar: 100 μm. (H) Representative fluorescence micrographs and quantification of EdU incorporation assays in H1299 cells across the indicated transfection groups as in (C). Scale bar: 100 μm. (I) Representative images and quantitative analysis of Transwell migration and Matrigel invasion assays in H1299 cells across the indicated transfection groups as in (A). Scale bar: 100 μm. (J) Representative images and quantitative analysis of Transwell migration and Matrigel invasion assays in H1299 cells across the indicated transfection groups as in (C). Scale bar: 100 μm. Data are presented as mean ± SD from three independent biological replicates (*n* = 3). Statistical comparisons across groups were evaluated by one‐way ANOVA with Tukey's post hoc test. *p < 0.05, **p < 0.01, ***p < 0.001, ****p < 0.0001.

Functionally, CCK‐8 proliferation assays revealed that PTEN knockdown abrogated the anti‐proliferative effect of NLRC4 overexpression, whereas PTEN restoration suppressed the accelerated proliferation caused by NLRC4 silencing (Figure 9E,F). EdU incorporation assays (Figure 9G,H) and Transwell migration/Matrigel invasion assays (Figure 9I,J) demonstrated that the inhibitory effects of NLRC4 on DNA synthesis, cell motility and invasiveness were largely reversed by PTEN depletion, while ectopic PTEN expression successfully rescued the aggressive oncogenic phenotypes induced by NLRC4 loss.

Collectively, these findings support a model wherein NLRC4 physically engages NFKB1 to restrain NF‐κB signalling and relieve PTEN repression, thereby inactivating the PI3K/AKT axis to licence protective autophagy and constrain LUAD progression.

### 3.7 NLRC4 Knockdown Accelerates Tumour Growth With Concurrent PTEN Loss and PI3K/AKT/mTOR Activation In Vivo

To validate whether the NLRC4/PTEN/PI3K/AKT axis operates in a physiologically relevant context in vivo, we established a subcutaneous xenograft model in female BALB/c nude mice. Mice were inoculated with PC9 cells stably expressing control shRNA (shNC) or two independent shRNAs targeting NLRC4 (shNLRC4‐1 and shNLRC4‐2; *n* = 5 mice per group).

At the experimental endpoint (28 days post‐injection), macroscopic examination of the excised xenografts revealed that tumours in both NLRC4‐knockdown groups were substantially larger than those in the shNC control group (Figure 10A). Dynamic tracking of tumour growth kinetics demonstrated that NLRC4 depletion markedly accelerated tumour progression throughout the 4‐week monitoring period (Figure 10B). In agreement with the growth curves, the final excised tumour weights were significantly elevated in the shNLRC4‐1 and shNLRC4‐2 cohorts compared to the shNC cohort (Figure 10C). Immunoblotting of harvested tumour tissues confirmed sustained knockdown of NLRC4 protein in vivo across both shRNA groups (Figure 10D).

**FIGURE 10:**
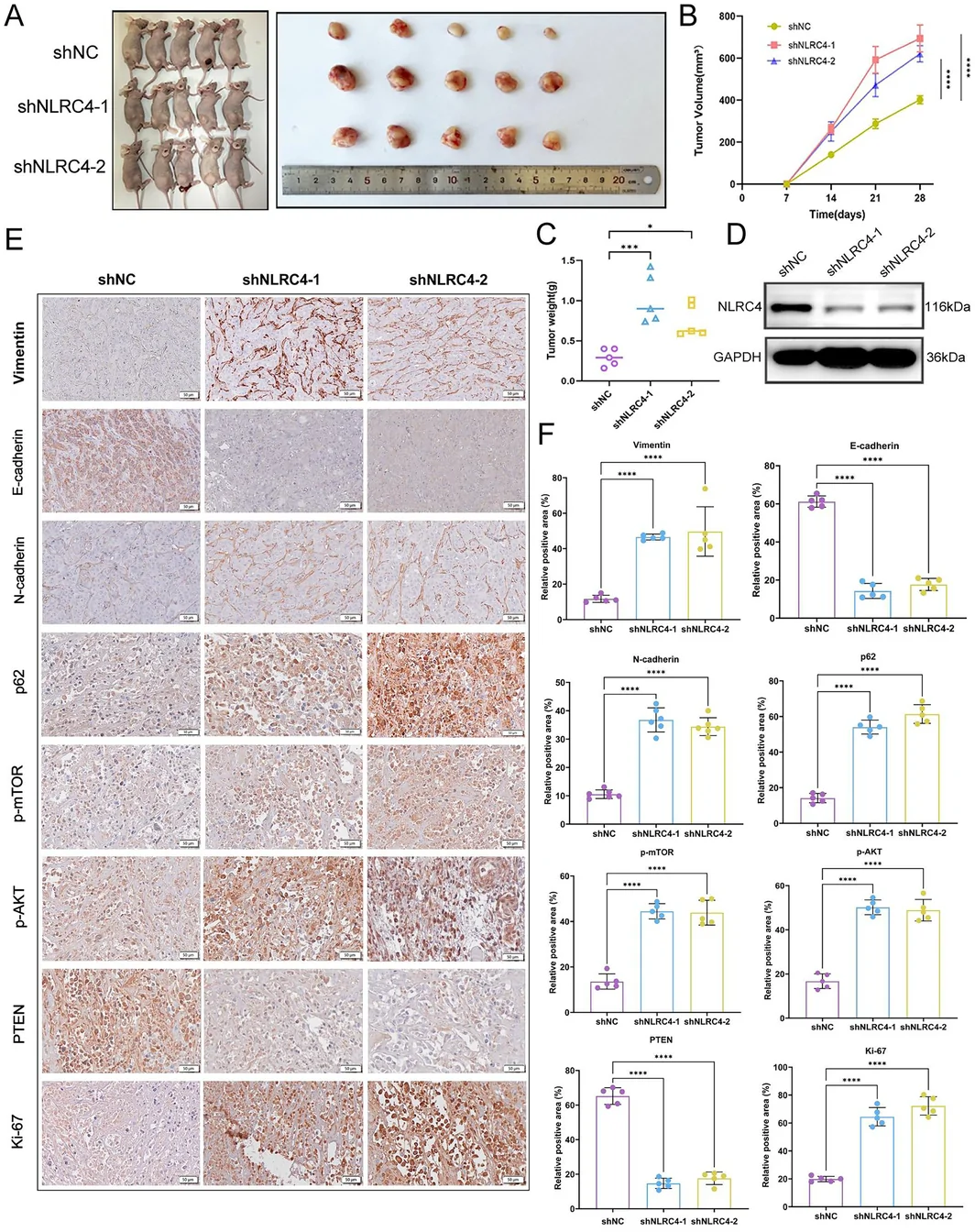
NLRC4 knockdown accelerates tumour growth with concurrent PTEN loss and PI3K/AKT/mTOR activation in vivo. (A) Macroscopic images of excised subcutaneous xenograft tumours harvested 28 days post‐injection. Female BALB/c nude mice (4 weeks old) were subcutaneously injected with PC9 cells (1 × 10^7^ cells/mouse) stably expressing shNC or two independent shRNAs targeting NLRC4 (shNLRC4‐1 and shNLRC4‐2; *n* = 5 mice per group). Tumours were harvested 28 days post‐injection. (B) Tumour growth kinetics. Tumour volumes were measured weekly using digital callipers and calculated as: Volume = (length × width^2^) × 0.5. Data represent mean ± SEM (*n* = 5 mice per group). (C) Final tumour weights measured at the experimental endpoint. Each dot represents an individual tumour (*n* = 5). (D) Western blot analysis of NLRC4 protein expression in harvested xenograft tumour tissues across the three treatment groups. (E) Representative immunohistochemistry (IHC) staining of xenograft tumour sections for NLRC4, PTEN, p‐AKT, p‐mTOR, p62, Ki‐67 and EMT markers (E‐cadherin, N‐cadherin, Vimentin). Scale bar: 50 μm. (F) Quantitative analysis of IHC staining across the three treatment groups (shNC, shNLRC4‐1, shNLRC4‐2). Positive cell percentages were calculated from five randomly selected high‐power fields per tumour section (*n* = 5 tumours per group). Data are presented as mean ± SD (or mean ± SEM for growth curves). Statistical comparisons among the three groups were evaluated by one‐way ANOVA with Tukey's post hoc test. *p < 0.05, **p < 0.01, ***p < 0.001, ****p < 0.0001.

To determine whether PTEN suppression, PI3K/AKT/mTOR pathway hyperactivation and autophagy blockade drive this accelerated tumour growth in vivo, we performed IHC analysis on xenograft sections. IHC staining confirmed robust downregulation of NLRC4 alongside a marked reduction in PTEN expression in NLRC4‐knockdown tumours compared with control tissues (Figure 10E,F). Concurrently, NLRC4‐depleted xenografts exhibited substantially elevated levels of p‐AKT and p‐mTOR, which were accompanied by a significantly higher proportion of Ki‐67‐positive proliferating cells (Figure 10E,F). Furthermore, we observed marked accumulation of the autophagy substrate p62 in NLRC4‐knockdown tumours (Figure 10E,F), indicating impaired autophagic flux in vivo. EMT marker analysis revealed decreased E‐cadherin expression alongside elevated N‐cadherin and Vimentin levels in NLRC4‐depleted tumours (Figure 10E,F).

Taken together, these in vivo findings seamlessly mirror our in vitro data, demonstrating that NLRC4 deficiency accelerates LUAD progression through concurrent PTEN loss, PI3K/AKT/mTOR pathway hyperactivation and autophagy suppression.

In conclusion, our integrated clinical, cellular, molecular and animal findings establish a coherent non‐canonical tumour‐suppressive paradigm: NLRC4 physically engages NFKB1 to relieve NF‐κB‐mediated transcriptional repression of PTEN, thereby tonically dampening the PI3K/AKT/mTOR axis to licence protective autophagy and constrain LUAD progression (Graphical Abstract).

## 4 Discussion

In this study, we delineate a non‐canonical, tumour‐intrinsic role for the cytosolic sensor NLRC4 in LUAD, identifying it as an upstream tumour suppressor that restrains malignant progression by licensing protective autophagy through suppression of the PI3K/AKT/mTOR signalling axis (Graphical Abstract).

Through clinical assessments and multi‐cohort transcriptomic analyses, we demonstrate that NLRC4 downregulation correlates with advanced clinical stages, lymph node metastasis in public cohorts and poor overall survival, while matched institutional tissue validation confirms its significant association with advanced TNM stage and pleural invasion (Figure 1; Figures S1–S4). Functionally, restoring NLRC4 expression attenuates LUAD cell proliferation, motility, invasion and EMT in vitro (Figure 4), whereas its depletion accelerates tumour growth and metastasis in vivo (Figure 10). Mechanistically, our pharmacological and genetic rescue experiments establish that the tumour‐suppressive activity of NLRC4 is functionally contingent upon intact autophagic machinery (Figures 5 and 6; Figure S7), and is mediated through the spatial restraint of NFKB1, which relieves transcriptional repression of PTEN to dampen PI3K/AKT signalling (Figures 7, 8, 9).

Autophagy exerts complex, context‐dependent functions during tumorigenesis, functioning to suppress early malignant transformation while frequently being co‐opted to sustain survival, metabolic adaptation and chemoresistance in established malignancies [19, 20, 21]. In LUAD, aberrant autophagic turnover is intimately linked to disease aggressiveness and therapeutic refractoriness [22, 23, 24]. While canonical views emphasize the cytoprotective, pro‐survival role of autophagy in advanced cancers, our findings indicate that basal or restored NLRC4 expression in LUAD cells triggers a protective autophagic programme that actively restricts oncogenic fitness. In particular, we observed that NLRC4‐driven autophagy coincides with the upregulation of E‐cadherin and the concomitant suppression of mesenchymal drivers (N‐cadherin, Vimentin and Snail), which is consistent with prior reports demonstrating that autophagic degradation of EMT‐associated transcription factors or signalling intermediates can impair metastatic dissemination [25, 26]. That pharmacological inhibition of autophagy with chloroquine largely abrogated NLRC4‐mediated suppression of migration and invasion suggests that, in this cellular context, autophagic flux acts predominantly as a tumour‐constraining rather than a tumour‐promoting mechanism.

A central conceptual finding of this study is the functional divergence of NLRC4 from its canonical inflammasome platform in LUAD epithelial cells. Classically, NLRC4 operates in myeloid lineages as a pattern‐recognition receptor that recruits upstream NAIP sensors and the ASC adaptor to assemble classical inflammasomes upon sensing bacterial ligands, triggering Caspase‐1 maturation, IL‐1β/IL‐18 processing and lytic pyroptosis [10, 11, 12]. However, emerging evidence indicates that several NLR family members display functional promiscuity, executing ‘moonlighting’ or scaffold duties in non‐immune cells independent of inflammasome assembly [14, 16]. Indeed, this non‐canonical paradigm is exemplified by NLRC3, which operates in non‐myeloid epithelial compartments to directly associate with PI3K and restrain PI3K/AKT/mTOR signalling to suppress colorectal tumorigenesis in an inflammasome‐independent manner [15]. Our computational analyses revealed that NLRC4 expression in LUAD is preferentially wired to core autophagy networks rather than canonical inflammasome or pyroptotic signatures (Figure 2; Figure S5). Experimentally, ectopic NLRC4 expression failed to provoke Caspase‐1 activation, Gasdermin cleavage (GSDMD/GSDME) or membrane permeabilization in LUAD cells (Figure 3J,K). Crucially, genetic or pharmacological blockade of autophagy (siATG5 or CQ) largely abolished NLRC4‐mediated anti‐tumour phenotypes (Figure 5; Figure S7). This phenotypic rescue strongly argues that protective autophagy, rather than inflammasome‐mediated pyroptotic lysis, acts as the primary functional effector of NLRC4 in LUAD epithelial cells.

A fundamental question arising from our findings is why the NLRC4‐NFKB1 interaction operates robustly in LUAD cells without causing a generalized shutdown of essential NF‐κB signalling in normal tissues. We propose that this non‐canonical interaction is governed by context‐dependent molecular competition, post‐translational modifications (PTMs) and stoichiometric buffering. First, in canonical myeloid lineages, NLRC4 is predominantly engaged by upstream NAIP sensors and the ASC adaptor to assemble classical inflammasomes upon ligand recognition [10, 12]. In contrast, in non‐myeloid LUAD epithelial cells where inflammasome machinery is naturally deficient, ‘unoccupied’ NLRC4 is uniquely positioned to engage alternative binding partners [15, 16]. Second, comparative Co‐IP assays revealed a markedly stronger NLRC4‐NFKB1 interaction in A549 cells than in normal bronchial epithelial BEAS‐2B cells (Figure 8E). This disparity is likely driven by the transformed tumour microenvironment (e.g., hypoxia, chronic metabolic stress and hyperactive oncogenic kinases), which may induce specific PTMs (such as phosphorylation) that alter NLRC4 conformation or subcellular localization [27, 28], thereby unmasking its binding interface for the Rel Homology Domain (RHD) of NFKB1 (p105/p50). Third, NLRC4 in LUAD cells does not completely abolish NF‐κB activity; rather, it functions as a cytoplasmic molecular sponge that fine‐tunes the stoichiometric pool of translocating p50/p65 heterodimers. This calibrated spatial restraint is sufficient to relieve NF‐κB‐mediated repression at the PTEN promoter while preserving baseline survival signalling, thereby establishing a tumour‐selective regulatory node that restrains malignant phenotypes without eliciting overt cellular collapse.

At the molecular level, our findings provide a plausible explanation for how NLRC4 coordinates the inhibition of the hyperactive PI3K/AKT/mTOR axis [29, 30, 31, 32]. Cross‐database analyses and institutional patient samples validated a synchronized expression pattern and robust positive correlation between NLRC4 and the tumour suppressor PTEN (Figures 1A–D and 8G,H). As PTEN is a major lipid phosphatase that antagonizes PI3K activation [9], and NF‐κB has been shown to transcriptionally repress PTEN [18], our identification of the direct physical interaction between NLRC4 and NFKB1 (p105/p50) provides a mechanistic bridge linking these pathways. We propose a working model wherein NLRC4 physically engages the NFKB1 complex in the cytoplasm, acting as an anchor that restrains p65 phosphorylation and nuclear translocation. This spatial constraint alleviates NF‐κB‐mediated transcriptional repression at the PTEN promoter, leading to restored PTEN expression and reinstatement of the inhibitory brake on the PI3K/AKT/mTOR cascade. Consequently, loss of NLRC4 in LUAD relieves this cytoplasmic sequestration, unleashing NF‐κB‐dependent PTEN silencing and subsequent pathway hyperactivation. Importantly, our bilateral genetic rescue experiments demonstrate that PTEN knockdown blocks NLRC4‐mediated autophagic activation and tumour suppression, while PTEN restoration rescues the oncogenic consequences of NLRC4 depletion, establishing PTEN as a crucial downstream mediator of this axis.

Several limitations of the study warrant acknowledgement. First, while our Co‐IP, expression and functional rescue data strongly support the NLRC4–NFKB1–PTEN axis, direct occupancy of NFKB1 at the PTEN promoter and the precise transcriptional kinetics following NLRC4 modulation require further elucidation via chromatin immunoprecipitation (ChIP) and promoter‐reporter assays. Second, our multi‐cohort transcriptomic evaluations largely relied on bulk RNA‐sequencing data, where signals could be partially confounded by tumour‐infiltrating stromal and immune cells; nevertheless, direct Western blot validation in paired clinical tissues and consistent phenotypes across multiple cell lines harbouring distinct driver mutations (A549, H1975, H1299, PC9) support a tumour‐intrinsic role for NLRC4, which could be further resolved using single‐cell RNA sequencing in future cohorts. Third, although our in vitro data demonstrate that LUAD cells bypass classical pyroptosis due to altered inflammasome stoichiometry, in vivo xenograft studies were performed in immunodeficient nude mice; prospective studies utilizing immunocompetent syngeneic models are necessary to evaluate whether NLRC4 expression in tumour cells influences the surrounding immune landscape, particularly regarding antigen presentation and anti‐tumour T cell immunity. Finally, prospective clinical cohorts will be essential to establish whether NLRC4 expression levels can serve as a predictive stratification biomarker for patients receiving PI3K/AKT/mTOR pathway inhibitors.

In conclusion, our study establishes a previously unappreciated tumour‐suppressive axis in LUAD, wherein NLRC4 acts through a non‐canonical, inflammasome‐independent mechanism to engage NFKB1, relieve PTEN repression and suppress PI3K/AKT/mTOR signalling, thereby licensing protective autophagy and restraining malignant progression. From a clinical perspective, assessing NLRC4 expression via immunohistochemistry or transcriptomic assays in biopsy specimens may provide valuable prognostic stratification and identify patient subsets with hyperactivated PI3K signalling who are most likely to benefit from targeted pathway inhibitors. From a therapeutic standpoint, strategies aimed at restoring or mimicking NLRC4 scaffold activity—such as developing small‐molecule stabilizers of NLRC4 or exploring targeted proteolysis‐targeting chimeras (PROTACs) directed against NFKB1—represent intriguing avenues for future drug discovery, particularly in combinatorial regimens paired with autophagy modulators to circumvent therapeutic resistance in lung adenocarcinoma.

## Author Contributions

**Yuxin Zhang:** writing – original draft, conceptualization, methodology, software, validation. **Shuangteng Liu:** methodology, validation, visualization. **Rui Li:** software, formal analysis, conceptualization. **Mengyu Zhang:** conceptualization, investigation, methodology, software. **Guang Yang:** investigation, validation. **Xinmiao Zhu:** investigation, validation. **Yiqing Qu:** writing – review and editing, funding acquisition, resources, project administration.

## Funding

This work was supported by the Natural Science Foundation of Shandong Province (Grant No. ZR2023QH397 to Y.Q.), the Major Project of National Natural Science Foundation of China (Grant No. 72293582 to Y.Q.) and the ECCM Program of Clinical Research Center of Shandong University (Grant No. 2021SDUCRCB001 to Y.Q.).

## Ethics Statement

This study was approved by the Medical Ethics Committee of Qilu Hospital of Shandong University (approval no. KYLL‐2021(KS)‐521). The animal study protocol was approved by the Institutional Animal Care and Use Committee of Qilu Hospital, Shandong University (Approval No. IACUC DWLL‐2024‐310).

## Consent

The authors are accountable for all aspects of the work, ensuring that questions related to the accuracy or integrity of any part of the work are appropriately investigated and resolved. All participants provided written informed consent both for participation in the study and for the publication of their anonymized data. The purpose, procedures and potential risks or benefits of the study were explained in detail to all participants prior to obtaining their consent. The consent forms explicitly included permission to publish anonymized research data. All data presented in this manuscript have been anonymized to ensure that no individual can be identified.

## Conflicts of Interest

The authors declare no conflicts of interest.

## Supporting information


**Figure S1:** Screening for autophagy‐related genes (ARGs) and construction of a prognostic signature.
**Figure S2:** Prognostic value of the risk score and its association with clinical traits.
**Figure S3:** NLRC4 is downregulated in LUAD and correlates with poor prognosis.
**Figure S4:** NLRC4 retains prognostic significance irrespective of EGFR or KRAS mutation status in TCGA‐LUAD.
**Figure S5:** Correlation profiles between NLRC4 and gene signatures associated with autophagy, pyroptosis and canonical inflammasomes in LUAD.
**Figure S6:** Expression profiling and genetic manipulation of NLRC4 in LUAD cell models.
**Figure S7:** Genetic blockade of autophagy via siATG5 abolishes the tumour‐suppressive effects of NLRC4 in LUAD cells.
**Table S1:** The siRNA and shRNA sequences used in this study.
**Table S2:** List of primary antibodies.
**Table S3:** List of 30 differentially expressed autophagy‐related genes in LUAD identified by TCGA transcriptome analysis.

## Data Availability

Publicly available datasets were analysed in this study. This data can be found here: The Cancer Genome Atlas (TCGA) database (https://portal.gdc.cancer.gov/) and the Gene Expression Omnibus (GEO) database (https://www.ncbi.nlm.nih.gov/geo/). The original contributions presented in the study are included in the article/Supporting Information. Further inquiries can be directed to the corresponding author.
