# Supplementary material for: NLRC4 Acts as a Non‐Canonical Tumour Suppressor in Lung Adenocarcinoma by Inhibiting PI3K/AKT/mTOR Signalling to Induce Protective Autophagy
Source: J Cell Mol Med. 2026 Oct 26;30(20):e71420. doi: 10.1111/jcmm.71420 (PMC13698827; doi:10.1111/jcmm.71420)
Supplement: Supplementary file 1 [file JCMM-30-e71420-s001.docx]

**List of Supporting Information**

Supporting Information 1: Supplementary Table 1

Supporting Information 2: Supplementary Table 2

Supporting Information 3: Supplementary Table 3

Supporting Information 4: Supplementary Figure 1

Supporting Information 5: Supplementary Figure 2

Supporting Information 6: Supplementary Figure 3

Supporting Information 7: Supplementary Figure 4

Supporting Information 8: Supplementary Figure 5

Supporting Information 9: Supplementary Figure 6

Supporting Information 10: Supplementary Figure 7

**Supplementary Table 1. The siRNA and shRNA sequences used in this study.**

|  | Sense | Antisense |
| --- | --- | --- |
| si-NLRC4-1 | 5′-GCGAAGAAGUAAACAUCAUTT-3′ | 5′-AUGAUGUUUACUUCUUCGCTT-3′ |
| si-NLRC4-2 | 5′-GGACAAUAGCCGAGCCCUUTT-3′ | 5′-AAGGGCUCGGCUAUUGUCCTT-3′ |
| si-NLRC4-3 | 5′-CCUGUAACCUCUUUCUUAATT-3′ | 5′-UUAAGAAAGAGGUUACAGGTT-3′ |
| si-ATG5 | 5′-GACGUUGGUAACUGACAAATT-3′ | 5′-UUUGUCAGUUACCAACGUCTT-3′ |
| si-PTEN | 5′-GACCUUAGUACUACUUUAATT-3′ | 5′-UUAAAGUAGUACUAAGGUCTT-3′ |
| siNC | 5′-UUCUCCGAACGUGUCACGUTT-3′ | 5′-ACGUGACACGUUCGGAGAATT-3′ |

**Supplementary Table 2. List of primary antibodies.**

| **Target** | **Catalog** | **Company** | **Dilution** |
| --- | --- | --- | --- |
| NLRC4 | A24336 | Abclonal | 1:1000 |
| P62 | T55546 | Abmart | 1:10000 |
| Beclin-1 | Ab302669 | Abcam | 1:20000 |
| LC3B | T55992 | Abmart | 1:2000 |
| PI3K | 4249T | Cell Signaling Technology | 1:1000 |
| AKT | 4685T | Cell Signaling Technology | 1:1000 |
| mTOR | 2972S | Cell Signaling Technology | 1:1000 |
| p-PI3K | 17366T | Cell Signaling Technology | 1:1000 |
| p-AKT | 4060S | Cell Signaling Technology | 1:2000 |
| E-cadherin | 3195T | Cell Signaling Technology | 1:400 |
| N-cadherin | 13116T | Cell Signaling Technology | 1:1000 |
| Vimentin | 5741T | Cell Signaling Technology | 1:1000 |
| Snail | 3879T | Cell Signaling Technology | 1:1000 |
| GAPDH | 10494-1-AP | Proteintech | 1:5000 |
| PTEN | 9559T | Cell Signaling Technology | 1:1000 |
| p-mTOR | 5536T | Cell Signaling Technology | 1:1000 |
| p-mTOR | 2976T | Cell Signaling Technology | 1:100 |
| NFKB1 | 13586T | Cell Signaling Technology | 1:1000 |
| p-p65 | AP1294 | ABclonal Technology | 1:10000 |
| Total p65 | A19653 | ABclonal Technology | 1:5000 |
| Caspase-1 | 2225T | Cell Signaling Technology | 1:1000 |
| Cleaved Caspase-1 | 89332T | Cell Signaling Technology | 1:1000 |
| Gasdermin D | 39754T | Cell Signaling Technology | 1:1000 |
| Cleaved Gasdermin D | 10137T | Cell Signaling Technology | 1:1000 |
| Gasdermin E | 19453T | Cell Signaling Technology | 1:1000 |
| Cleaved Gasdermin E | 55879T | Cell Signaling Technology | 1:1000 |
| Flag-tag (Anti-Flag) | 20543-1-AP | proteintech | 1:20000 |
| HA-tag (Anti-HA) | 66006-2-Ig | proteintech | 1:20000 |
| ASC (PYCARD) | 67824T | Cell Signaling Technology | 1:1000 |
| IgG | 30000-0-AP | proteintech | 1:1000 |
| ATG5 | 12994T | Cell Signaling Technology | 1:1000 |
| Ki67 | 9449T | Cell Signaling Technology | 1:800 |

**Supplementary Table 3. List of 30 differentially expressed autophagy-related genes in LUAD identified by TCGA transcriptome analysis.**

| **Gene** | **log2**  **(Fold Change)** | **P-value** | **Adjusted P-value**  **(FDR)** | **Expression Trend** |
| --- | --- | --- | --- | --- |
| IKBKE | 1.88 | 0.00000067 | 0.0002 | Upregulated |
| BIRC5 | 2.31 | 0.00000018 | 0.0003 | Upregulated |
| VMP1 | 1.67 | 0.00000032 | 0.0004 | Upregulated |
| ERO1A | 1.64 | 0.00000074 | 0.0005 | Upregulated |
| ITGB4 | 1.63 | 0.00000083 | 0.0006 | Upregulated |
| FOS | -1.56 | 0.00000093 | 0.0005 | Downregulated |
| HSPB8 | -1.41 | 0.0000015 | 0.001 | Downregulated |
| DAPK2 | -1.45 | 0.0000018 | 0.001 | Downregulated |
| CCL2 | -1.37 | 0.0000024 | 0.001 | Downregulated |
| EIF4EBP1 | 1.53 | 0.0000027 | 0.002 | Upregulated |
| NLRC4 | -1.52 | 0.0000032 | 0.002 | Downregulated |
| GRID1 | -1.44 | 0.0000037 | 0.002 | Downregulated |
| ERBB2 | 1.47 | 0.0000041 | 0.003 | Upregulated |
| IFNG | 1.42 | 0.0000056 | 0.004 | Upregulated |
| DAPK1 | -1.47 | 0.0000059 | 0.004 | Downregulated |
| BNIP3 | 1.38 | 0.0000062 | 0.004 | Upregulated |
| NRG1 | -1.39 | 0.000011 | 0.002 | Downregulated |
| ATIC | 1.35 | 0.000012 | 0.002 | Upregulated |
| CX3CL1 | -1.23 | 0.000021 | 0.003 | Downregulated |
| NRG3 | -1.33 | 0.000029 | 0.005 | Downregulated |
| CDKN2A | 1.22 | 0.000038 | 0.006 | Upregulated |
| ATG9B | 1.27 | 0.000043 | 0.007 | Upregulated |
| PRKCQ | -1.32 | 0.000045 | 0.008 | Downregulated |
| MAP1LC3C | -1.22 | 0.000048 | 0.008 | Downregulated |
| DLC1 | -1.25 | 0.000054 | 0.009 | Downregulated |
| DRAM1 | -1.28 | 0.000067 | 0.011 | Downregulated |
| PPP1R15A | -1.18 | 0.000072 | 0.012 | Downregulated |
| TMEM74 | 1.19 | 0.000078 | 0.013 | Upregulated |
| P4HB | 1.31 | 0.000086 | 0.014 | Upregulated |
| PTK6 | 1.29 | 0.000091 | 0.015 | Upregulated |

LUAD, lung adenocarcinoma; TCGA, The Cancer Genome Atlas; FDR, false discovery rate.

**Supplementary Figure 1****. Screening for autophagy-related genes (ARGs) and construction of a prognostic signature.**

(A) Heatmap showing the expression profiles of autophagy-related genes in the TCGA-LUAD cohort (n=539 tumors vs. 59 normal samples). (B) Volcano plot of differentially expressed autophagy-related genes. (C) Box plots comparing the expression of autophagy-related genes between normal and tumor tissues (evaluated by Mann-Whitney U test). (D) Univariate Cox regression analysis of autophagy-related genes associated with overall survival (HR, 95% CI; log-rank test). (E) Multivariate Cox regression analysis identifying the final five-gene prognostic signature (HR, 95% CI; P=0.002). (F) Kaplan-Meier survival curves comparing overall survival between high- and low-risk groups (log-rank test, P<0.001). (G) Time-dependent ROC curve for the 1-year overall survival prediction (AUC = 0.744). (H) Univariate Cox regression and (I) multivariate Cox regression analyses of the risk score alongside clinical covariates. Data are presented as HR (95% CI).

**Supplementary Figure 2. Prognostic value of the risk score and its association with clinical traits.**

(A) Distribution of risk scores for patients stratified by the six-gene signature. (B) Scatter plot of patient survival status versus risk score. (C) Heatmap of expression levels for the six prognostic ARGs. (D-I) Correlation analysis between ARG expression/risk scores and clinical characteristics (age, sex, stage, TNM stage); only significant results (P < 0.05) are visualized.

**Supplementary Figure 3. NLRC4 is downregulated in LUAD and correlates with poor prognosis.**

(A, B) NLRC4 mRNA expression across diverse cancer types (TIMER 2.0) and in LUAD (GEPIA 2.0). (C) NLRC4 mRNA downregulation in TCGA-LUAD tumor tissues compared to matched normal tissues. (D) Kaplan-Meier survival curve for overall survival in the TCGA-LUAD cohort. (E–H) Validation of the prognostic value of NLRC4 in four independent GEO cohorts (GSE50081, GSE31210, GSE11969, and GSE68465). (I–K) Association of NLRC4 expression with (I) histological subtypes, (J) lymph node metastasis, and (K) clinical stage in the TCGA-LUAD cohort. *P < 0.05, **P < 0.01, ***P < 0.001, ****P < 0.0001; ns, not significant.

**Supplementary Figure 4. NLRC4 retains prognostic significance irrespective of EGFR or KRAS mutation status in TCGA-LUAD.**

Kaplan-Meier survival curves comparing overall survival (OS) between NLRC4-high and NLRC4-low groups (stratified by median expression) in LUAD patients categorized by driver mutation status: (A) EGFR-mutant (n=76), (B) EGFR-wild-type (n=451), (C) KRAS-mutant (n=142), and (D) KRAS-wild-type (n=385). P-values were calculated using the log-rank test.

**Supplementary Figure 5. Correlation profiles between NLRC4 and gene signatures associated with autophagy, pyroptosis, and canonical inflammasomes in LUAD.**

(A, B) Scatter plots illustrating the correlation between NLRC4 mRNA expression and core autophagy-related genes, including (A) ATG5 and (B) BECN1, in the TCGA-LUAD dataset. (C–F) Scatter plots illustrating the correlation between NLRC4 mRNA expression and Gasdermin family members, including (C) GSDMB, (D) GSDMC, (E) GSDMD, and (F) GSDME. (G) Scatter plots showing the correlation between NLRC4 and canonical inflammasome components (IL1B, PYCARD/ASC, and CASP1). Spearman’s correlation coefficients (R) and corresponding P-values are indicated in each panel. Transcripts per million (TPM) was used for mRNA expression quantification.

**Supplementary Figure 6. Expression profiling and genetic manipulation of NLRC4 in LUAD cell models.**

(A) NLRC4 mRNA expression across human LUAD cell lines extracted from the Cancer Cell Line Encyclopedia (CCLE) database. (B) Representative immunohistochemistry (IHC) staining of NLRC4 in normal lung versus LUAD tissues obtained from the Human Protein Atlas (HPA). (C, D) Baseline NLRC4 expression evaluated by RT-qPCR (C) and Western blot (D) across a panel of LUAD cell lines compared to normal bronchial epithelial cells (BEAS-2B). (E, F) Western blot validation of NLRC4 overexpression (OE) efficiency in A549 and H1299 cells (E), and siRNA-mediated knockdown (siNLRC4) efficiency in H1299 and H1975 cells (F). GAPDH was used as the loading control for Western blots. Data are presented as mean ± SD from three independent biological replicates (n=3). Statistical comparisons were evaluated by two-tailed Student's t-test or one-way ANOVA with Tukey's post-hoc test. *P < 0.05, **P < 0.01, ***P < 0.001, ****P < 0.0001; ns, not significant.

**Supplementary Figure 7. Genetic blockade of autophagy via siATG5 abolishes the tumor-suppressive effects of NLRC4 in LUAD cells.**

(A) Western blot analysis of LC3B-I/II, p62, and ATG5 in H1299 and A549 cells transfected with the indicated combinations: Vector + siNC, NLRC4-OE + siNC, Vector + siATG5, or NLRC4-OE + siATG5. (B) Densitometric quantification of LC3B-II/I, p62, and ATG5 protein levels from (A). (C) Representative fluorescence micrographs of EdU incorporation assays across the indicated transfection groups. Scale bar: 100 μm. (D) Quantification of EdU-positive proliferating cells from (C). GAPDH was used as the loading control for Western blots. Data are presented as mean ± SD from three independent biological replicates (n=3). Statistical comparisons were evaluated by two-tailed Student's t-test or one-way ANOVA with Tukey's post-hoc test. *P < 0.05, **P < 0.01, ***P < 0.001, ****P < 0.0001; ns, not significant.
